# NETosis-Related Biomarkers in Systemic Lupus Erythematosus, Rheumatoid Arthritis, Psoriatic Arthritis and Ankylosing Spondylitis: A Comparative Study

**DOI:** 10.3390/ijms262412127

**Published:** 2025-12-17

**Authors:** Mark M. Melamud, Anna S. Tolmacheva, Alexey E. Sizikov, Nataliya A. Klyaus, Evgenii S. Zhuravlev, Grigory A. Stepanov, Georgy A. Nevinsky, Valentina N. Buneva, Evgeny A. Ermakov

**Affiliations:** 1 Institute of Chemical Biology and Fundamental Medicine, Siberian Branch of the Russian Academy of Sciences, 630090 Novosibirsk, Russia; marken94@mail.ru (M.M.M.); tolmacheva.anna0301@gmail.com (A.S.T.); alex.sizikov.as@gmail.com (A.E.S.); evgenijur@gmail.com (E.S.Z.); stepanovga@niboch.nsc.ru (G.A.S.); nevinsky@1bio.ru (G.A.N.); 2 Department of Rheumatology, Immunopathology Clinic, Research Institute of Fundamental and Clinical Immunology, Siberian Branch of the Russian Academy of Sciences, 630099 Novosibirsk, Russia; 3 Department of Faculty Therapy with the Clinic, Almazov National Medical Research Centre, 197341 Saint Petersburg, Russia; klyausn@mail.ru; 4 Department of Natural Sciences, Novosibirsk State University, 630090 Novosibirsk, Russia

**Keywords:** NETosis, cell-free DNA, myeloperoxidase, citrullinated histone H3, IL-18, systemic lupus erythematosus, rheumatoid arthritis, psoriatic arthritis, ankylosing spondylitis, biomarkers

## Abstract

NETosis is assumed to be involved in the pathogenesis of common rheumatic diseases such as systemic lupus erythematosus (SLE), rheumatoid arthritis (RA), psoriatic arthritis (PsA), and ankylosing spondylitis (AS). However, the levels of circulating NETosis biomarkers and the extent of changes in these markers in specific rheumatic diseases are not fully understood. In this study, cell-free DNA (cfDNA) concentration as a non-specific marker, as well as myeloperoxidase (MPO) and citrullinated histone H3 (H3cit) as specific markers of NETosis, were investigated in SLE, RA, PsA, and AS. Analysis of covariance, accounting for sex, age and disease duration, showed that total cfDNA was elevated in SLE and AS compared with healthy subjects. Nuclear and mitochondrial cfDNA were elevated in four diseases. However, nuclear cfDNA was increased to a greater extent in SLE but mitochondrial cfDNA was higher in RA. MPO and H3cit were significantly elevated in SLE compared with other diseases, although MPO was also higher in RA. Elevated concentrations of MPO and H3cit in SLE were associated with the presence of concomitant cardiovascular diseases. The effect of biological therapy on mitochondrial cfDNA, MPO, and H3cit was also shown. The proinflammatory cytokine IL-18, implicated in the induction of NETosis, was similarly elevated in the four rheumatic diseases. Thus, the most striking signs of NETosis are found in SLE, although they are also present in RA. PsA and AS were mainly characterized by an increase in cfDNA. These data highlight characteristic changes in NETosis markers in four rheumatic diseases.

## 1. Introduction

Rheumatic diseases (RD) represent a serious medical and social problem. Numerous studies show that the prevalence of rheumatic diseases such as rheumatoid arthritis (RA) [1], systemic lupus erythematosus (SLE) [2], ankylosing spondylitis (AS) [3], and psoriatic arthritis (PsA) [4] is steadily increasing. The prevalence of RA is approximately 200 cases per 100,000 population, and according to prognostic studies, this prevalence may double by 2050 [5]. The prevalence of SLE varies depending on the region of the world and ranges from 3 to 517 cases per 100,000 population [6]. The prevalence of PsA is more than 100 cases per 100,000 population [7], and AS affects approximately 9–30 people per 100,000 population [8]. Some rheumatic diseases manifest in people at a young, working and reproductive age. For example, SLE more often affects young women [9], and AS more often affects men under 45 years of age [10]. Such patients increase the economic burden on healthcare systems [11]. Therefore, it is essential to look for novel potential pathogenic processes, diagnostic markers, or therapeutic approaches for rheumatic disorders.

Although RA, SLE, AS, and PsA have different pathogenetic mechanisms, they share a number of common features. All these diseases are characterized by dysregulation of both innate and adaptive immunity, the development of autoimmune reactions, and sterile inflammation. However, NETosis can also be considered a common mechanism for rheumatic pathologies. NETosis is a process in which neutrophils release into the extracellular space chromatin networks decorated with various proteins, also called neutrophil extracellular traps (NETs) [12]. NETosis, initially aimed at fighting pathogenic microorganisms, may also be associated with autoimmune inflammation. NETs consist mostly of highly immunogenic or chemically active molecules. The NET “scaffold” consists of DNA, histones, and other molecules that act as damage-associated molecular patterns (DAMPs) and attract immune cells to the lesion [13]. However, NETs are also decorated with molecules that directly destroy tissue. Myeloperoxidase (MPO) catalyzes the formation of hypochlorous, bromic, and hypophosphorous acids, which pathologically alter the structures of DNA, lipids, and proteins [14], while neutrophil elastase degrades collagen and other structural proteins [15].

Circulating NETosis markers are conventionally divided into specific and non-specific [16]. Circulating cell-free DNA (cfDNA) is considered a non-specific NETosis marker because it can be released during other types of cell death and by active secretion within exosomes and vesicles [17]. Specific circulating markers of NETosis include MPO, neutrophil elastase, and citrullinated histones (H3 is primarily studied) [16]. In addition, circulating MPO-DNA complexes may also indicate NET activation, although this marker is considered less valid because MPO-DNA complexes do not always correlate with other NET biomarkers in vivo [18].

A plethora of studies have linked NETosis processes to the pathogenesis of RA [19], SLE [20], AS [21], and PsA [22]. However, circulating markers of NETosis have not been comprehensively studied in these rheumatic diseases. For example, it is known that cfDNA concentrations increase in these rheumatic diseases [23,24,25,26]. However, the origin of cfDNA (nuclear or mitochondrial) is much less well understood, although nuclear and mitochondrial cfDNA differ in their immunostimulatory potential [27]. Specific markers of NETosis, MPO and citrullinated histone H3 (H3cit), have been well studied in SLE and RA [28,29,30,31,32,33], but less so in AS and PsA. For example, we were unable to find studies of circulating H3cit in AS. Additionally, it is quite interesting to compare multiple rheumatic diseases in a single experiment since presents an opportunity to highlight potential markers for differential diagnosis and discover distinctive alterations in each condition.

The aim of this study was to analyze the concentration of NETosis markers in four common rheumatic diseases, SLE, RA, AS, and PsA, compared with healthy subjects and between diseases. Non-specific (total, genomic, and mitochondrial cfDNA) and specific (MPO and H3cit) NETosis markers, as well as clinical associations, were examined in this study.

## 2. Results

### 2.1. Clinical Characteristics of Sample

A total of 223 people participated in the study: 44 patients with RA, 53 with AS, 30 with PsA, 23 with SLE and 73 healthy subjects. The clinical features of the participant groups are described in Table 1. The sex ratio differed significantly between groups. Specifically, all SLE patients were women, while there were more women among RA and PsA patients, and more men among AS patients. Furthermore, the age of RA and AS patients was higher than that of healthy controls. These differences were associated with the clinical and epidemiological features of the analyzed rheumatic diseases, in particular with the fact that SLE, RA, and PsA are more common in women [4,5,6], and AS in men [8], and that RA often develops at a later age (onset occurring at 60–70 years of age) than other rheumatic diseases [5]. Disease duration was also longer in AS than in SLE patients. Differences in age and disease duration were taken into account in further analysis.

Based on the DAS28 index, most RA patients had moderate disease activity (Table 1). Among RA patients, 80% and 82% were positive for rheumatoid factor (RF) and anti-citrullinated protein antibodies (ACPA), respectively. Among patients with AS, 76% carried the 27 allele of the B locus of the human leukocyte antigen gene (HLA-B27). Patients with AS mainly had low disease activity according to the ASDAS-CRP/ASDAS-ESR indices and moderate activity according to the BASDAI index. Most patients with PsA had moderate disease activity according to the DAS28 and DAPSA indices. Among patients with SLE, more than half had low disease activity. SLE patients had higher levels of anti-dsDNA antibodies than RA and AS patients and healthy individuals (Table 1).

It is important to note that all patients received therapy. Specifically, 56% of patients with RA, 79% with AS, and 47% with PsA received biological disease-modifying antirheumatic drugs (bDMARDs). The rest received synthetic disease-modifying antirheumatic drugs (csDMARDs) or glucocorticoids. Among bDMARDs, rituximab was the most common drug in RA (63% of those taking bDMARDs), but patients also took golimumab, certolizumab pegol, olokizumab, and sarilumab. AS patients received certolizumab pegol (28.5%), netakimab (19%), secukinumab (17%), adalimumab (14%), golimumab (12%), and ixekizumab (9.5%). In PsA, patients received certolizumab pegol (29%), secukinumab (29%), netakimab (21%), ixekizumab (14%), and guselkumab (7%). In case of csDMARDs, 43% of patients with RA, 32% with AS, 40% with PsA, and 8% with SLE received methotrexate. SLE patients received only csDMARDs, with hydroxychloroquine being the most common drug (71%). 88% of SLE patients, 23% of RA patients, 6% of AS patients took glucocorticoid drugs (prednisolone). PsA patients did not receive glucocorticoids.

### 2.2. Nonspecific Markers of NETosis: Total, Nuclear, and Mitochondrial cfDNA

**Figure 1:**
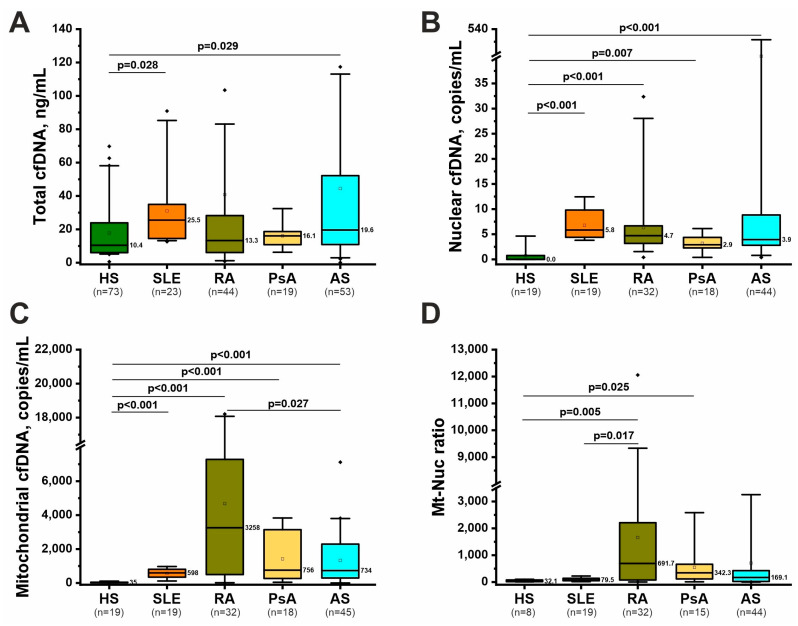
Total (**A**), nuclear (**B**), and mitochondrial (**C**) cfDNA concentration, as well as mitochondrial to nuclear (Mt-Nuc) cfDNA ratio (**D**) in plasma of patients with systemic lupus erythematosus (SLE), rheumatoid arthritis (RA), psoriatic arthritis (PsA), ankylosing spondylitis (AS) and healthy subjects (HS). Data are presented as box plots showing median values (line in the center of the box, numerical values are shown on the right), quartiles (boxes), 5–95% percentile ranges (whiskers), and values outside the boxplot range (black diamonds). The significance of differences was assessed using analysis of covariance (ANCOVA), accounting for sex, age and disease duration as covariates, with a post hoc test. Differences in Mt-Nuc cfDNA ratio were calculated by the Kruskal–Wallis test with Dunn’s post hoc test. Only significant *p*-values that passed Tukey’s correction for multiple comparisons are presented.

Circulating cfDNA is considered a non-specific marker of cell death, including NETosis [17]. Total cfDNA is an integral indicator of all circulating nucleic acids in exosomes, vesicles, and in complexes with DNA-binding proteins; however, due to the characteristics of the analytical method (fluorometric detection with an intercalating dye), it allows mainly double-stranded DNA (dsDNA) to be recorded [34]. The analysis showed that total cfDNA concentration varied significantly between groups (descriptive statistics are presented in Supplementary Table S1). Analysis of covariance (ANCOVA) with sex, age, disease duration, and group as covariates showed that sex, age and disease duration were not significant covariates in the ANCOVA model (Supplementary Table S2); therefore, these parameters did not significantly affect total cfDNA concentration. Post hoc analysis showed that total cfDNA concentration increased only in SLE and AS compared to healthy controls (Figure 1A and Supplementary Table S3). Estimated marginal means of total cfDNA concentration in the analyzed groups are presented in Supplementary Figure S1.

The pool of circulating nucleic acids includes DNA of nuclear and mitochondrial origin, which differ in inflammatory potential [35]. In this study, to more thoroughly characterize cfDNA, the concentration of nuclear and mitochondrial cfDNA was examined using digital PCR (Supplementary Tables S4–S7). ANCOVA revealed that age, sex, and disease duration were not significant covariates for both nuclear cfDNA and mitochondrial cfDNA (Supplementary Tables S4 and S6). Post hoc analysis revealed that nuclear cfDNA was elevated in all four rheumatic diseases compared to healthy individuals, with median cfDNA concentrations being highest in SLE (Figure 1B and Supplementary Table S5). However, no significant differences were found between the diseases, as also evident in the estimated marginal means plot (Supplementary Figure S2).

A different pattern was observed for mitochondrial cfDNA (Figure 1C). Mitochondrial cfDNA concentrations were elevated in four rheumatic diseases compared with healthy controls. However, the highest mitochondrial cfDNA levels were observed in RA, while the lowest levels were found in SLE (Figure 1C and Supplementary Table S7 and Figure S3). Mitochondrial cfDNA levels were also higher in RA than in AS. Analysis of the mitochondrial to nuclear (Mt-Nuc) cfDNA ratio showed a significant increase in RA and PsA compared with healthy controls (Figure 1D). The Mt-Nuc ratio was also elevated in RA compared with SLE.

Thus, nuclear and mitochondrial cfDNA increased in four rheumatic diseases, but total cfDNA increased significantly only in SLE and AS, and only an increasing trend was observed in RA and PsA (Figure 1). The obtained data indicate that the increase in cfDNA in SLE is associated mainly with an increase in nuclear DNA, while in RA, mitochondrial cfDNA increases to a greater extent. In PsA and AS, the contributions of nuclear and mitochondrial cfDNA are quite similar.

### 2.3. Specific Markers of NETosis: H3cit and MPO

**Figure 2:**
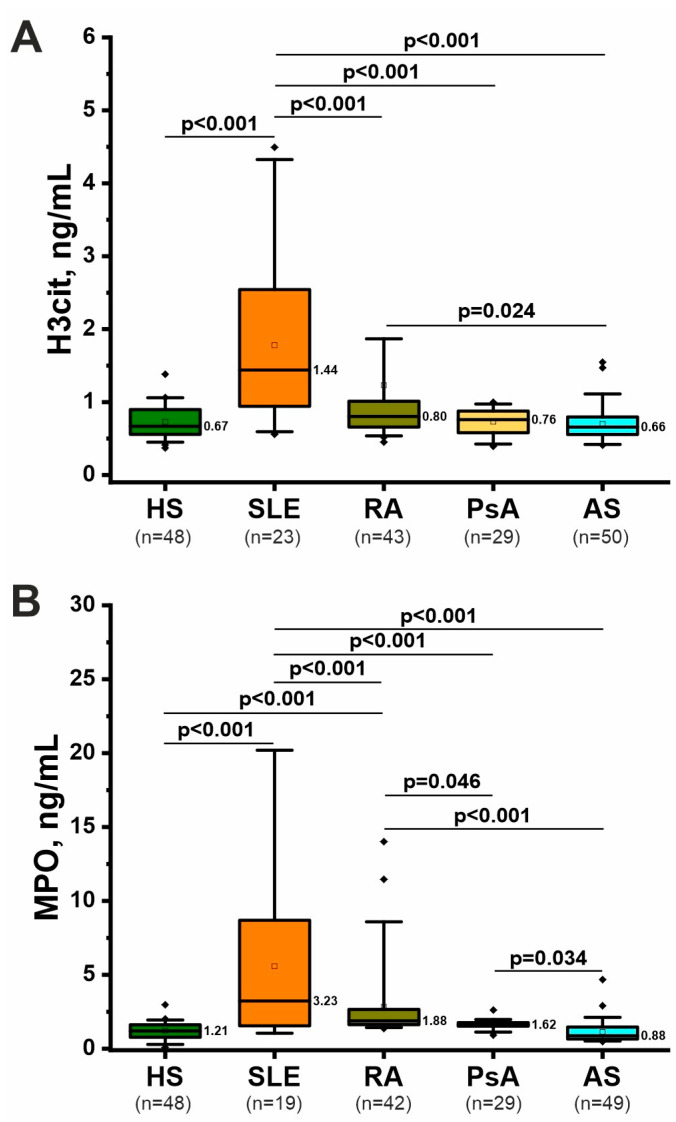
Concentration of specific NETosis biomarkers, citrullinated histone H3 (H3cit) (**A**) and myeloperoxidase (MPO) (**B**), in plasma of patients with rheumatic diseases and healthy subjects. The data are presented as boxplots similar to Figure 1. The significance of differences was assessed using ANCOVA, accounting for sex, age and disease duration as covariates, with a post hoc test. Only significant *p*-values that passed Tukey’s correction for multiple comparisons are presented.

H3cit and MPO are considered to be specific markers of NETosis [13]. ANCOVA showed that H3cit was elevated only in SLE but not in other diseases compared with healthy individuals (Figure 2A and Supplementary Tables S8 and S9). H3cit concentrations in SLE were higher than in RA, PsA, and AS. Thus, the highest H3cit levels were detected in SLE, which is also well illustrated by the estimated marginal means plot (Supplementary Figure S4).

MPO concentrations were elevated in SLE and RA compared with healthy controls (Figure 2B and Supplementary Tables S10 and S11), according to ANCOVA results. Notably, SLE patients had the highest MPO levels compared with other rheumatic diseases, which is also evident in the estimated marginal means plot (Supplementary Figure S5). MPO concentrations were higher in RA than in AS. MPO was also elevated in PsA compared with AS (Figure 2B).

Importantly, sex, age and disease duration were not significant covariates in the ANCOVA (Supplementary Tables S8 and S10), so these clinical factors did not significantly influence the obtained data.

Thus, specific NETosis markers, H3cit and MPO, were most significantly elevated in SLE. In RA, MPO is significantly elevated, but H3cit only tends to increase compared to healthy individuals (Figure 2).

### 2.4. Inflammatory Marker: IL-18

**Figure 3:**
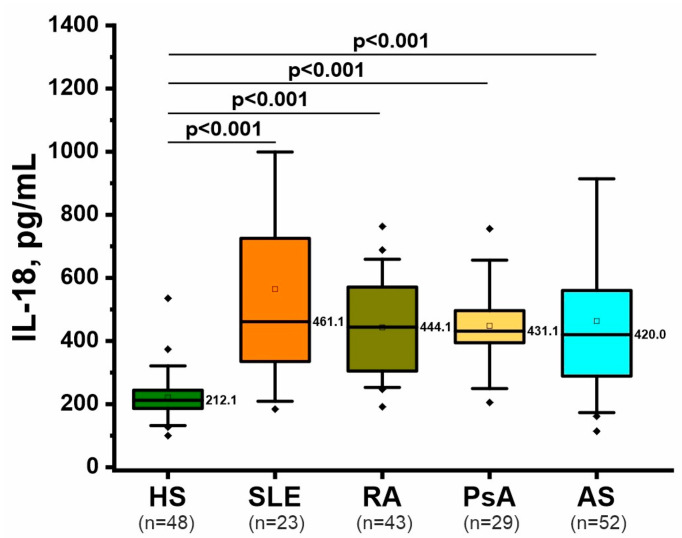
Concentration of the proinflammatory marker IL-18 in plasma of patients with rheumatic diseases and healthy subjects. The data are presented as boxplots similar to Figure 1. The significance of differences was assessed using ANCOVA, accounting for sex, age and disease duration as covariates, with a post hoc test. Only significant *p*-values that passed Tukey’s correction for multiple comparisons are presented.

IL-18 is a proinflammatory cytokine actively involved in the recruitment of neutrophils to the site of injury and the induction of NETosis [36]. IL-18 was increased to approximately the same extent in the four rheumatic diseases compared with healthy individuals and did not differ significantly between patients with rheumatic diseases (Figure 3, Supplementary Tables S12 and S13 and Figure S6). Age, sex, and disease duration were not significant predictors in the ANCOVA model (Supplementary Table S12). Thus, IL-18, a cytokine that promotes NETosis, is approximately equally elevated in the rheumatic diseases analyzed.

### 2.5. ROC Analysis and Combined NETosis-Associated Biomarker Profiles

Binary logistic regression models were constructed for groups with significant differences in NETosis markers. ROC analysis of the constructed models showed fairly high-performance metrics for classifying patients and healthy individuals (Table 2 and Supplementary Figure S7). For example, mitochondrial cfDNA has proven to be a virtually ideal marker for classifying SLE patients and healthy individuals. Nuclear cfDNA has emerged as a promising marker for differentiating AS or RA patients from healthy subjects. In the case of PsA, a model based on mitochondrial cfDNA demonstrated good metrics. The constructed models demonstrated lower performance metrics for classifying patients with various rheumatic diseases. Notably, total cfDNA had relatively low performance metrics. Thus, nonspecific markers such as nuclear and mitochondrial cfDNA demonstrated slightly higher metrics than the specific NETosis markers, MPO and H3cit, for classifying patients and healthy individuals. However, specific NETosis markers emerged as potential markers for differential diagnosis.

**Figure 4:**
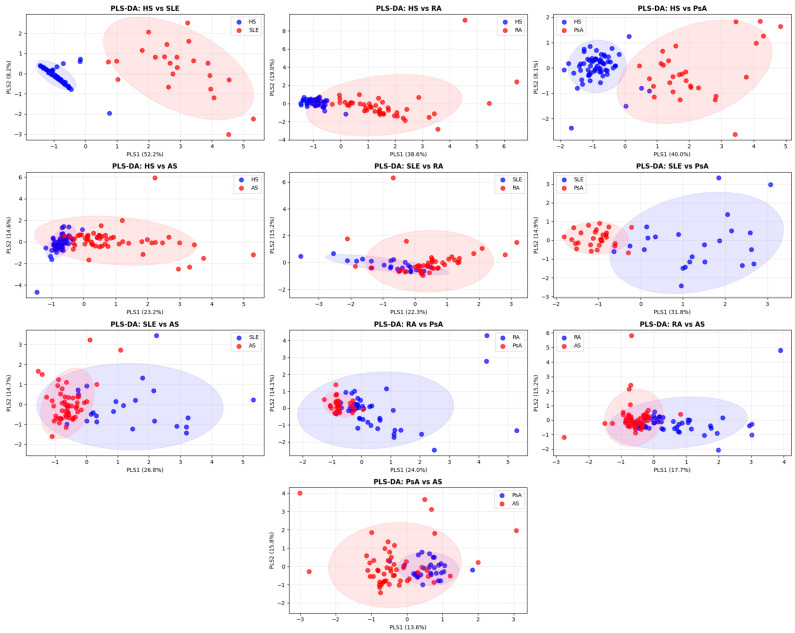
Partial Least Squares Discriminant Analysis (PLS-DA) plot reflecting combined NETosis-associated biomarker profiles. Pairwise comparison of profiles for all analyzed groups is presented. Each point represents an individual participant. Ellipses of 95% confidence interval are shown in the figures. Non-overlapping ellipses indicate good separation between samples. Conversely, overlapping ellipses indicate similarity between groups in NETosis marker levels.

Partial Least Squares Discriminant Analysis (PLS-DA) is a good tool to reduce the dimensionality of data and graphically demonstrate similarities or differences in the analyzed samples. Analysis of combined NETosis-associated biomarker profiles presented as PLS-DA plots showed that SLE and PsA patients differed quite significantly from healthy individuals, since the 95% confidence interval ellipses practically did not overlap (Figure 4). SLE and PsA patients also differed significantly in their combined NETosis-associated biomarker profiles. In other cases, the profiles of patients and healthy controls, as well as patients with various rheumatic diseases, overlapped significantly, indicating similar levels of NETosis markers.

### 2.6. Clinical Associations

Levels of NETosis markers may depend on the clinical characteristics of the diseases. Analysis of sex-dependent differences revealed that IL-18 concentrations in males diagnosed with RA were 1.6 times higher than in females (Supplementary Table S14). No significant sex-dependent differences were found in AS. Females with PsA had higher levels of the specific NETosis markers (MPO and H3cit), but lower levels of total cfDNA compared to males. Healthy females had higher concentrations of mitochondrial cfDNA.

Next, clinical associations for each disease were analyzed. SLE patients were divided into two groups based on their SELENA-SLEDAI score: (1) no/low activity (0–5) and (2) moderate/high/very high activity (>5). No significant differences were found in the markers studied. However, among patients with SLE, nine had various comorbid cardiovascular diseases (CVDs) (mainly arterial hypertension, for details see Section 4.1), while the remaining 14 patients did not have CVDs. Analysis of differences based on the presence of CVDs revealed a 3.3-fold increase in MPO and a 2-fold increase in H3cit in SLE patients with comorbid CVDs (Supplementary Table S15). Although this conclusion is based on a small sample size and the CVDs were quite heterogeneous, the contribution of comorbid CVDs to the increase in NETosis markers deserves further study.

NETosis markers did not differ significantly between RA patient subgroups according to the DAS28 index: ≤3.2—remission/low activity and >3.2—moderate/high activity.

In PsA, no significant differences were found in subgroups depending on the DAS28 index. However, patients with DAPSA > 14 (moderate/high activity) had higher levels of mitochondrial cfDNA (Supplementary Table S16).

AS patients with ASDAS-CRP > 2.1 (high/very high activity) had higher mitochondrial cfDNA levels (Supplementary Table S17), although no significant differences were found in the ASDAS-ESR subgroups. Meanwhile, patients with BASDAI > 3 (moderate activity) had higher MPO levels (Supplementary Table S18).

Analysis of NETosis marker levels in patients depending on the disease stage revealed an increase in MPO in RA patients and mitochondrial cfDNA in PsA patients in the active stage of the disease compared with those in remission (Supplementary Table S19). No differences were found in SLE and AS.

**Figure 5:**
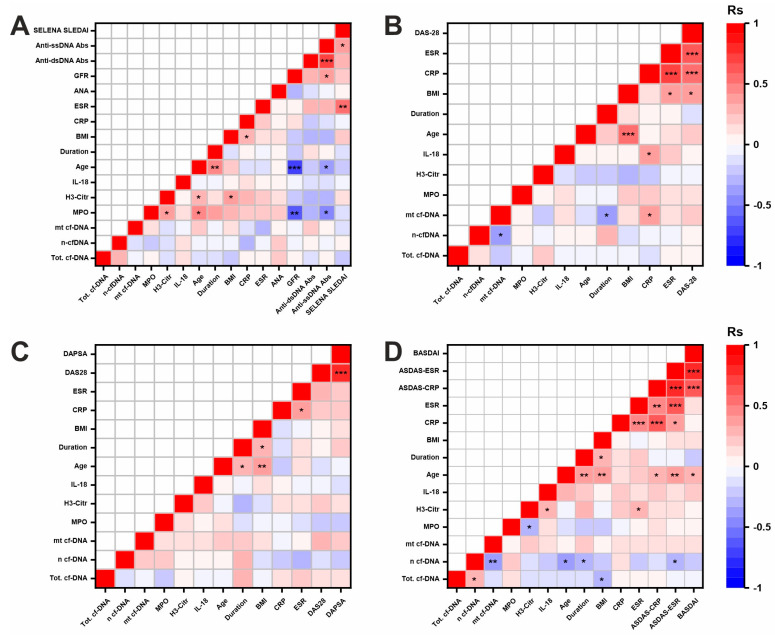
Correlation matrices reflecting the association of NETosis marker concentrations with clinical parameters in four study groups: (**A**) SLE, (**B**) RA, (**C**) PsA and (**D**) AS. Rs—Spearman correlation coefficient. *—0.01 < *p* < 0.05; **—0.001 < *p* < 0.01; ***—*p* < 0.001. Abbreviations: Anti-ssDNA Abs—antibodies to single-stranded DNA, Anti-dsDNA Abs—antibodies to double-stranded DNA, GFR—glomerular filtration rate, ANA—antinuclear antibodies. Other abbreviations have been given previously.

The results of the correlation analysis of NETosis marker levels with clinical parameters are presented in Figure 5.

The age of SLE patients was positively correlated with the level of MPO (Rs = 0.574, *p* = 0.01) and H3cit (Rs = 0.453, *p* = 0.03) (Figure 5A). BMI was positively correlated with H3cit (Rs = 0.431, *p* = 0.04). The level of MPO was significantly inversely correlated with the glomerular filtration rate (Rs = −0.756, *p* = 1.8 × 10^−6^), indicating an association with kidney damage in SLE.

In RA, a negative correlation between mitochondrial cfDNA and disease duration was found (Rs = −0.38, *p* = 0.03) (Figure 5B). CRP levels correlated positively with IL-18 (Rs = 0.401, *p* = 0.01) and mitochondrial cfDNA (Rs = 0.404, *p* = 0.02), indicating a link between mitochondrial cfDNA levels and overall inflammation.

No significant correlation associations were identified in PsA (Figure 5C).

In AS, nuclear cfDNA levels were negatively correlated with age (Rs = −0.353, *p* = 0.02), disease duration (Rs = −0.297, *p* = 0.05), and ASDAS-ESR (Rs = −0.324, *p* = 0.03). In addition, H3cit levels were positively correlated with IL-18 (Rs = 0.283, *p* = 0.04) and ESR (Rs = 0.323, *p* = 0.025), indicating a link between inflammation and H3cit levels in AS (Figure 5D).

Multiple regression analysis showed that NETosis markers are not significant predictors of DAS28 in RA (Supplementary Table S20). Only CRP was a significant predictor in the regression model. A similar pattern was observed in AS. Only CRP was a significant predictor of ASDAS-CRP (Supplementary Table S21), and only ESR was a significant predictor of ASDAS-ESR (Supplementary Table S22), but not NETosis markers in AS. Models for other diseases were not significant.

Finally, the observed changes in NETosis markers could be related to the patients’ treatment. To account for the effect of treatment, patients were divided into two subgroups: (1) those receiving bDMARDs and (2) csDMARDs or glucocorticoid drugs. Diagnosis was considered as a secondary variable. A two-way ANOVA showed that diagnosis, as well as the diagnosis/treatment combination, had a significant effect on mitochondrial cfDNA (Table 3). For H3cit, only the diagnosis/treatment combination significantly affected this marker. A direct effect of treatment, as well as the diagnosis/treatment combination, was shown for MPO. These data highlight the impact of therapy on NETosis markers in rheumatic diseases.

To more thoroughly analyze the impact of therapy, NETosis marker levels were compared in specific RDs. The analysis showed that mitochondrial cfDNA levels in RA patients receiving bDMARDs were 8 times lower than in those receiving csDMARDs or glucocorticoids (Supplementary Table S23). In PsA patients receiving bDMARDs, MPO levels were significantly reduced (Supplementary Table S23). No significant differences were found in AS and SLE. Because glucocorticoids can affect neutrophils, NETosis marker levels were also assessed in patients taking prednisone compared to patients taking other medications. However, no significant differences were found.

## 3. Discussion

### 3.1. Characteristic Changes in NETosis Markers in Four Rheumatic Diseases

NETosis has long been associated with the pathogenesis of RA [19], SLE [20], AS [21], and PsA [22]. However, the magnitude of changes in NETosis biomarkers in different rheumatic diseases is not entirely clear. This work revealed strong evidence of NETosis in SLE and, to a lesser extent, in RA (Figure 1, Figure 2 and Figure 3). In AS and PsA, only non-specific signs of NETosis were observed, in particular an increase in cfDNA (Figure 1). Previous studies have well-documented changes in cfDNA in rheumatic diseases, although the data are somewhat contradictory. In particular, some studies have shown increased [24,37,38] or decreased [39] levels of total cfDNA in RA. However, in the case of SLE, the results on increased total cfDNA are more consistent [23,24,40]. Studies on AS and PsA are few, but they also indicate increased total cfDNA [25,41]. Therefore, the results of this study on an increase in total cfDNA in SLE and AS are consistent with the literature (Figure 1). However, this study did not detect an increase in total cfDNA in RA and PsA, although the median values were higher than in controls (Figure 1).

The origin of cfDNA (nuclear or mitochondrial) in RDs is much less well understood. The origin of cfDNA is important because mitochondrial cfDNA is a proinflammatory agent due to its similarity to bacterial DNA and often appears in the extracellular space during vital NETosis. In contrast, nuclear cfDNA is less active as a DAMP and accumulates during suicidal NETosis [13]. Existing experimental data on nuclear and mitochondrial cfDNA in RDs are contradictory. In SLE and RA, both an increase [42,43,44] and no changes in the level of nuclear cfDNA [45,46] have been shown. Data in SLE indicate an increase [45], decrease [42], or no changes in mitochondrial cfDNA [43]. In RA, there is evidence of increased [46] and no changes in mitochondrial cfDNA [44]. In the case of AS and PsA, we were unable to find data on nuclear and mitochondrial cfDNA, so the results of this study fill this gap in knowledge.

Taken together, the data on increased cfDNA in rheumatic diseases are a non-specific marker of NETosis (Figure 1). It is worth considering that cfDNA can be released during other types of cell death and also actively secreted within exosomes and vesicles [17].

H3cit is considered a more specific marker of NETosis because citrullination of histones by the enzyme peptidyl arginine deaminase 4 (PAD4) occurs in neutrophils during NETosis [13], although PAD4 can citrullinate histones during other cellular processes. An increase in this marker has been shown in SLE [33] and RA, and the H3cit level correlated with RA activity [32]. A recent study showed an increase in circulating H3cit in treatment-naive or csDMARD-only PsA patients [47]. No similar studies on circulating H3cit levels could be found in AS.

MPO is also considered a marker of NETosis, but it is less specific than H3cit because circulating MPO may be associated with other types of neutrophil death (necrosis/apoptosis). There is evidence that MPO concentration increases in RA [28,29] and SLE [30,31]. Moreover, SLE is even known to have a complication such as MPO-specific antineutrophil cytoplasmic antibody (ANCA) vasculitis [48]. Therefore, the data obtained in this study are consistent with the literature (Figure 2). In PsA, an increase in the concentration of both MPO [49] and MPO-DNA complexes [22] is recorded. There is evidence of an increase in MPO activity, but not the concentration of this enzyme, in AS patients [50]. This study failed to confirm an increase in MPO levels in PsA and AS, which may be due to sample characteristics and, primarily, the effect of treatment (see below).

IL-18 is a cytokine that reflects the level of proinflammatory state and can directly initiate NETosis [36]. The concentration of this cytokine in the blood has also been studied in RA [51], SLE [52], AS, and PsA [53], but research on this topic is relatively limited. The results of this study confirm a proinflammatory state and elevated IL-18 levels in rheumatic diseases (Figure 3).

Correlation and regression analyses revealed no association between NETosis markers and DAS28, ASDAS-CRP/ASDAS-ESR, SELENA-SLEDAI, or DAPSA (Figure 5 and Supplementary Tables S20–S22). This result may be explained by treatment effects (discussed below). Taken together, these data do not support the role of NETosis as a marker of clinical activity in treated cohorts.

### 3.2. Effect of Therapy on NETosis Markers

This study identified the effect of bDMARDs on mitochondrial cfDNA, MPO, and H3cit levels (Table 3). Analysis of individual diseases revealed a decrease in mitochondrial cfDNA in RA and H3cit in PsA (Supplementary Table S23). Therefore, the above-described data on the insignificant differences in MPO and H3cit levels in AS and PsA, and the increase in MPO but not H3cit in RA (Figure 2), may be associated with the effectiveness of bDMARDs. It is worth considering that 79% of patients with AS, 47% with PsA, and 56% of patients with RA received bDMARDs. At the same time, no significant effect of prednisone was revealed. However, due to the small sample size, we were unable to conduct a more detailed analysis of the effects of specific drugs in different rheumatic diseases. Currently, there is evidence that bDMARDs therapy helps reduce NETosis markers in RA, AS, and PsA [54,55,56]. Therefore, further studies are needed to elucidate the effects of bDMARDs and csDMARDs on NETosis markers and to identify potential markers of therapy response.

### 3.3. Association of NETosis with CVD and Glomerular Filtration Rate in SLE

Although the detected increase in MPO and H3cit levels in SLE patients with CVD (mainly with arterial hypertension) is based on a relatively small sample size (Supplementary Table S15), these results indicate the involvement of NETosis in the pathogenetic mechanisms of comorbid cardiovascular pathology and NET-driven inflammation in SLE. A large body of data indicates a link between NETosis and the development of CVD, therefore elevated NETosis markers in rheumatic diseases can be considered as a predisposing factor for the development of cardiovascular pathology [57]. Activated neutrophils and NETosis products, in particular H3cit, contribute to endothelial dysfunction and impaired vascular relaxation, thereby triggering the development of arterial hypertension [58]. A recent study also showed that dsDNA dose-dependently reduces the cellular viability of human umbilical vein endothelial cells, suggesting that elevated cfDNA levels in SLE are associated with endothelial dysfunction [59]. In addition, some autoantibodies can trigger NETosis, also contributing to endothelial damage [60]. Furthermore, NET remnants can also promote inflammation and the production of proinflammatory cytokines, further contributing to the development of CVD [61]. There is already evidence linking elevated levels of proinflammatory cytokines with comorbid CVD in SLE [62,63]. Overall, NET-driven inflammation may be associated with the development of CVD in SLE and other rheumatic diseases [64].

This study also found an inverse correlation between MPO levels and glomerular filtration rate (Rs = −0.756, *p* = 1.8 × 10^−6^) in SLE (Figure 5). These findings are intriguing given that SLE patients with lupus nephritis were not included in the study. There is evidence that some NETosis products promote thrombosis, endothelial damage including tubuloepithelial cell necrosis, disrupt intracellular junctions, promote vascular leakage and fibrosis, thereby contributing to dysfunction of the glomerular filtration barrier [65]. The data from this study indicate the need to monitor renal damage in SLE patients even without a diagnosis of lupus nephritis.

### 3.4. Limitations

The studied markers may be associated not only with NETosis but also with other cell death pathways. The division into non-specific (cfDNA) and specific (MPO and H3cit) markers of NETosis is rather arbitrary. It should be noted that cfDNA can be released during the death of not only neutrophils but also other cells. MPO can appear in the blood during other neutrophil death processes (necrosis/apoptosis). Although H3cit is more specific for NETosis, histone citrullination by PAD4 can also occur during other cellular processes [66]. The lack of correlation of MPO and H3cit with cfDNA may also indicate the contribution of cell death processes other than NETosis, since during NETosis MPO and H3cit are usually released in complex with DNA (Figure 5). Other limitations of this work include the relatively small sample size, sex ratio imbalance, and differences in age and disease duration. However, ANCOVA controlling for sex, age, and disease duration revealed no significant effects of these covariates on NETosis markers (please see Supplementary Tables S2, S4, S6, S8, S10 and S12). However, further research into the influence of sex on NETosis markers is needed in light of evidence that sex-related genetic factors may modulate autoimmunity [67]. Furthermore, NETosis markers can be significantly influenced by therapy (Table 3 and Supplementary Table S23), so more careful analysis of the effects of specific drugs in RDs is needed. Due to the cross-sectional design, the study did not account for the dynamic nature of the markers and their characteristic time-dependent changes. It is likely that NETosis markers will change significantly over the course of the disease and during treatment. The identified association of NETosis markers with CVD in SLE was based on a relatively small sample size and requires confirmation in subsequent studies. Complete blood count data were not collected during participant recruitment, so this study did not examine associations of NETosis marker levels with the total neutrophil count.

## 4. Materials and Methods

### 4.1. Recruitment of Patients and Healthy Individuals and Clinical Status Assessment

Participants were recruited between July 2023 and July 2025. The study included patients with the following diagnoses: RA according to the 2010 American College of Rheumatology/European League Against Rheumatism (ACR/EULAR) criteria [68]; AS (or axial spondyloarthritis) according to the New York criteria and the criteria of the Assessment of Spondyloarthritis International Society (ASAS) [69,70]; PsA according to the Psoriatic Arthritis Classification Criteria (CASPAR) [71]; SLE according to the 2019 ACR/EULAR diagnostic criteria [72]. The inclusion criteria for patients were as follows: consent to participate in the study; age over 18 years; verified diagnosis of RA, PsA, SLE, or AS. The exclusion criteria were as follows: the presence of other autoimmune diseases; active oncological diseases; severe liver and kidney damage; acute inflammatory diseases two weeks before blood sampling; surgeries within the last two months; HIV infection; pregnancy. SLE patients with lupus nephritis were also excluded. The inclusion criteria for healthy individuals were consent to participate, age over 18 years, and the absence of somatic pathology. Patient recruitment and anamnesis collection were performed by qualified rheumatologists at the Institute of Fundamental and Clinical Immunology (Novosibirsk, Russia). Healthy donors were recruited at the Institute of Chemical Biology and Fundamental Medicine (Novosibirsk, Russia).

The clinical status of patients with RA was assessed using the Disease Activity Score-28 (DAS28) [73]. Radiologic stages of RA were evaluated according to the recommendations of Steinbroker et al. [74]. AS patients were assessed using the AS disease activity score (ASDAS-CRP and ASDAS-ESR) and Bath AS disease activity index (BASDAI) [75,76]. In case of PsA, the patient’s status was assessed using the DAS28 and disease activity in psoriatic arthritis (DAPSA) [73,77]. For SLE, the Safety of Estrogens in Lupus Erythematosus National Assessment-SLE Disease Activity Index (SELENA-SLEDAI) was used [78].

SLE patients were divided into two subgroups based on the presence of concomitant CVD, as in our previous study [63]. CVD was diagnosed in accordance with international (European Society of Cardiology) and national (Russian Society of Cardiology) guidelines [79,80,81]. Among CVD, arterial hypertension was the most common (89%), but there were also patients with congestive heart failure, cardiomyopathy, angina, arrhythmias, atherosclerosis, and coronary heart disease. One third of patients had one CVD, but some patients had two or three concomitant CVDs.

Patients were also divided into subgroups based on their clinical index scores. According to the SELENA-SLEDAI score, SLE patients were divided into the following groups: <5—no–low activity and >5—moderate–high–very high activity. RA patients were divided into two subgroups according to the DAS28 index: ≤3.2—remission/low activity and >3.2—moderate/high activity. AS patients were divided into two subgroups according to the ASDAS-CRP and ASDAS-ESR: <2.1—no/low activity and ≥2.1—high/very high activity. AS patients were divided into two subgroups according to the BASDAI index: <3—low activity and 4–6—moderate activity (there were only two patients with BASDAI > 6 corresponding to high activity, but they were not taken into account in the analysis). PsA patients according to the DAS28 were divided into two subgroups: ≤3.2—remission/low activity and >3.2—moderate/high activity. PsA patients were also divided into two subgroups according to the DAPSA index: ≤14—remission/low activity and >14—moderate/high activity.

### 4.2. Obtaining Blood Plasma for Analysis

Blood from participants was collected into K3-EDTA vacuum tubes (Cat. #: 455036, VACUETTE, Greiner Bio-One GmbH, Kremsmünster, Austria). A two-step centrifugation protocol was used to obtain plasma according to previously published recommendations [82]. Blood was first centrifuged for 15 min at 2000× *g* at 4 °C in an Eppendorf 5804R refrigerated centrifuge (Cat #: 5805000017, Eppendorf, Hamburg, Germany). Plasma was then transferred to a new tube (15 mL) and centrifuged a second time for 10 min at 5000× *g* at 4 °C. The resulting platelet-free plasma was aliquoted and stored at −20 °C until further analysis.

### 4.3. Isolation of Total cfDNA from Plasma

Total cfDNA was isolated from plasma using the D-blood-250 kit (Biolabmix LLC, Novosibirsk, Russia) according to the manufacturer’s recommendations. Briefly, the kit is based on the selective adsorption of nucleic acids from a pre-lysed sample (in the presence of proteinase K) on a silica membrane, followed by washing and elution of the purified product. Total cfDNA was isolated from an equal volume of plasma (200 μL) from each participant. The concentration of isolated total cfDNA was determined by fluorometric detection using the Qubit dsDNA High Sensitivity Assay Kit (Cat. # Q32854, Thermo Fisher Scientific, Dreieich, Germany) on a Qubit 4 fluorimeter (Thermo Fisher Scientific, Dreieich, Germany).

### 4.4. Analysis of Nuclear and Mitochondrial cfDNA by Digital PCR

The QIAcuity One digital PCR system (Qiagen, Hilden, Germany) was used to analyze nuclear and mitochondrial cfDNA. The human ribonuclease P (*RP*) gene was used as a reference gene for nuclear DNA detection (Table 4). The RP gene product is widely distributed in eukaryotic cells, has a single copy, is often used as an internal/positive control in multiplex PCR systems, and is recommended by the Centers for Disease Control and Prevention of the United States [83]. The NADH dehydrogenase subunit 1 (*MT-ND1*) gene was used as a second reference gene for mitochondrial DNA detection (Table 4), similar to [84]. Each gene-specific probe was labeled with a different fluorescent dye to ensure multiplexing and simultaneous analysis of two genes.

Due to the limited volume of total cfDNA and limited space on PCR plates, participant samples were randomly selected for analysis. The reaction was carried out using the QIAcuity Probe PCR Kit (Cat. # 250102, Qiagen, Hilden, Germany), which contains all the necessary components for PCR. The following final concentrations of each of the two primers were used: 0.3 µM forward primer, 0.3 µM reverse primer, and 0.15 µM probe. Preliminary experiments were performed using special 24-well digital PCR plates QIAcuity Nanoplate 26k 24-well (Cat. # 250001, Qiagen, Hilden, Germany) with 26,000 microwells in each well. In these experiments, the required sample volume of isolated cfDNA for optimal detection of mitochondrial and genomic cfDNA was determined. For the screening experiment to analyze cfDNA in all samples, 96-well QIAcuity Nanoplate 8.5k 96-well digital PCR plates (Cat. # 250021, Qiagen, Hilden, Germany) with 8500 microwells in each well were used. Thermal cycling conditions were as follows: pre-cycling heat activation of DNA polymerase at 95 °C (3 min) followed by 40 cycles of denaturation at 95 °C (15 s) and combined annealing/extension at 60 °C (30 s). The analysis of the results was conducted using a QIAcuity Software Suite (v2.2) (Qiagen, Hilden, Germany) and presented in the following concentration units: copies/mL.

### 4.5. H3cit, MPO, IL-18 Analysis by ELISA

H3cit concentration in plasma was determined by ELISA using a Citrullinated Histone H3 (Clone 11D3) ELISA Kit (Cat. # 501620, Cayman Chemical, Ann Arbor, MI, USA). The lower limit of quantification was 0.3 ng/mL. The measurement range was 0.15–10 ng/mL. Blood plasma for analysis was pre-diluted 3-fold with PBS. Measurements were carried out in accordance with the manufacturer’s recommendations. The intra-assay coefficient of variation (CV) was 3.3% and inter-assay CV was 5.6%.

MPO concentration was measured using the Uncoated Human MPO (Myeloperoxidase) ELISA Kit (Cat. # E-UNEL-H0048, Elabscience, Houston, TX, USA). This kit allows varying the concentrations of capture antibodies, detection antibodies, and horseradish peroxidase (HRP) conjugate to increase sensitivity. In this study, the following dilutions were used: capture antibodies (1:500), biotinylated detection antibodies (1:500), and streptavidin-HRP conjugate (1:500). An Ancillary Reagent Kit (Cat. # E-ELIR-K001, Elabscience, Houston, TX, USA) containing all necessary solutions for the assay was also used. The measurement range was 0.78–50 ng/mL. Blood plasma for analysis was pre-diluted 3-fold with PBS. The remaining stages of the analysis were performed in accordance with the manufacturer’s recommendations. The intra-assay CV was 4.7% and inter-assay CV was 11.7%.

IL-18 concentration was determined using the Interleukin-18-EIA-BEST ELISA Kit (Cat. # 8770, Vector-Best, Novosibirsk, Russia). Plasma was not diluted before analysis. The lower limit of quantification was 2 pg/mL. The measurement range was 0–1000 pg/mL. Measurements were carried out in accordance with the manufacturer’s recommendations. The intra-assay CV was 10.2% and inter-assay CV was 10.6%.

ELISA results were recorded on a Multiskan™ FC Microplate Photometer (Thermo Fisher Scientific Inc., Waltham, MA, USA) at a wavelength of 450 nm.

### 4.6. Statistical Analysis

Statistical analysis was performed primarily using the free and open-source statistical platform Jamovi (version 2.6.44, Sydney, Australia). OriginPro 2021 (OriginLab Corporation, Northampton, MA, USA) was used for data visualization. The Shapiro–Wilk test was used to determine the normality of data distribution in individual samples. Since the data generally did not follow a normal distribution, the results are presented as medians and interquartile range (Q1–Q3) unless otherwise stated. NETosis marker concentrations were compared between samples using ANCOVA, accounting for sex, age, and disease duration as covariates, with a post hoc test and Tukey’s correction for multiple comparisons (implemented in Jamovi). Before ANCOVA, the data were normalized using the natural logarithm transformation: Ln (x + 1). Differences in subgroups were assessed using the Mann–Whitney test or the Kruskal–Wallis test with Dunn’s post hoc test (in the case of more than two groups). Categorical variables were assessed using the chi-square test with Yates’s correction. Correlations were assessed using the Spearman test. Multiple linear regression was used to assess the relationship between the dependent variable (clinical scales) and predictors (NETosis markers). Before conducting multiple regression, the data were normalized using the natural logarithm transformation: Ln (x + 1). Missing values were excluded in all analyses. Partial Least Squares Discriminant Analysis (PLS-DA) was used to reduce data dimensionality. PLS-DA was implemented in the Google Colab code processing environment (https://colab.research.google.com/ accessed on 10 November 2025) using NumPy 2.3.3, Pandas 2.3.0, Matplotlib 3.10.6, and Seaborn 0.13.2 libraries in the Python 3.13.0 programming language. Binary logistic regression and ROC analysis was performed in OriginPro 2021 (OriginLab Corporation, Northampton, MA, USA). A two-way ANOVA was used to examine the effects of two factors (disease and treatment) and their combination on NETosis markers. The two-way ANOVA was implemented using the NumPy 2.3.3, Pandas 2.3.0, and Matplotlib 3.10.6 libraries in the Python 3.13.0 programming language.

## 5. Conclusions

This study confirmed that the most prominent features of NETosis are observed in SLE. However, other diseases, particularly RA, are also associated with NETosis activation. AS and PsA demonstrated primarily an increase in cfDNA, a non-specific marker of NETosis. The observed changes in NETosis markers may be related to the effects of bDMARDs therapy, but longitudinal studies are needed to confirm the effects of bDMARDs in rheumatic diseases. The association of elevated MPO and H3cit levels with CVD is intriguing and merits further investigation. Taken together, these data highlight the diverse roles of NETosis in rheumatic diseases.

## Figures and Tables

**Table 1:** Clinical features of patients and healthy individuals.

Feature ^1^	RA (*n* = 44) (1)	AS (*n* = 53) (2)	PsA (*n* = 30) (3)	SLE (*n* = 23) (4)	HS (*n* = 73) (5)	*p*-Value ^2^
Sex (M/F), %	25/75	69/31	30/70	0/100	30/70	<0.01
Age, years	49.5 ± 12.6	44.9 ± 12.1	45.1 ± 12.9	44.1 ± 16.0	37.8 ± 12.8	1 vs. 5: <0.001 2 vs. 5: <0.02
Disease duration, years	8 (4.75–15)	12 (8–22)	10 (3–21)	6 (3–12)	–	2 vs. 4: <0.01
BMI	26 (23–28)	26 (21–28)	26 (21–30)	25 (21–28)	23 (20–26)	1 vs. 5: <0.02
ESR, mm/h	2.6 (0.8–11.3)	2.8 (1.1–6.2)	4.3 (2.4–9.8)	2.2 (0.8–8.9)	–	0.19
CRP, mg/L	14.5 (6.5–28)	10 (4–18)	16 (10–26)	15 (8.5–38)	–	0.5
Anti-dsDNA antibodies, IU/mL	2.6 (0.67–5.2)	1.8 (0.88–3.9)	–	21.4 (8.8–117)	0.45 (0–1.8)	1 vs. 4: <0.001; 1 vs. 5: <0.02; 2 vs. 4: <0.001 4 vs. 5: <0.0001
Clinical index	DAS28: 3.4 (2.6–4.5)	ASDAS-CRP: 1.7 (1.3–2.5); ASDAS-ESR: 1.8 (1.31–2.6); BASDAI: 1.9 (1.0–3.2)	DAS28: 3.5 (1–9.4); DAPSA: 14 (6–22)	SELENA-SLEDAI: 4 (2–8)	–	–

^1^ Data are presented as median (Q1–Q3) or mean ± standard deviation. ^2^ Differences in categorical variable values were determined using the chi-square test with Yates’s correction. Differences in continuous variable values were analyzed using the Kruskal–Wallis test with Dunn’s post hoc test. Abbreviations: BMI—body mass index, ESR—erythrocyte sedimentation rate, CRP—C-reactive protein, Anti-dsDNA antibodies—antibodies to double-stranded DNA, DAS28—disease activity score-28, ASDAS—ankylosing spondylitis disease activity score, DAPSA—disease activity in psoriatic arthritis, SELENA-SLEDAI—safety of estrogens in lupus erythematosus national assessment—systemic lupus erythematosus disease activity index.

**Table 2:** Results of ROC curve analysis of NETosis-associated markers.

Biomarker	Comparison	AUC (95%CI)	*p*-Value	Cut-Off	Sensitivity (95%CI)	Specificity (95%CI)	Youden’s J
Tot. cfDNA	SLE–HS	0.75 (0.62–0.89)	3.1 × 10^−4^	11.9	53 (40–67)	100 (88–100)	0.53
Nuc. cfDNA	SLE–HS	0.98 (0.94–1)	4.8 × 10^−7^	2.66	90 (78–95)	100 (88–100)	0.9
Mt. cfDNA	SLE–HS	1 (1–1)	1.4 × 10^−7^	115	100 (93–100)	100 (88–100)	1
MPO	SLE–HS	0.88 (0.8–0.96)	1.3 × 10^−6^	2.04	98 (89–100)	74 (56–86)	0.72
H3cit	SLE–HS	0.85 (0.75–0.94)	2.4 × 10^−6^	0.94	85 (73–93)	78 (61–89)	0.64
IL-18	SLE–HS	0.91 (0.94–0.98)	3 × 10^−8^	325	96 (86–99)	83 (65–92)	0.78
Tot. cfDNA	AS–HS	0.67 (0.58–0.77)	9.1 × 10^−4^	9.9	48 (35–62)	83 (66–93)	0.31
Nuc. cfDNA	AS–HS	0.93 (0.87–0.99)	5.9 × 10^−8^	1.6	90 (78–95)	93 (78–98)	0.83
Mt. cfDNA	AS–HS	0.86 (0.71–1)	4.7 × 10^−6^	124	100 (93–100)	82 (62–92)	0.82
IL-18	AS–HS	0.87 (0.79–0.95)	2.5 × 10^−10^	274	88 (75–94)	81 (63–91)	0.68
Nuc. cfDNA	RA–HS	0.95 (0.89–1)	1.1 × 10^−7^	1.53	90 (78–95)	97 (83–100)	0.86
Mt. cfDNA	RA–HS	0.94 (0.84–1)	1.8 × 10^−7^	221	100 (93–100)	94 (79–98)	0.94
MPO	RA–HS	0.87 (0.8–0.94)	1.6 × 10^−9^	11.4	63 (48–75)	98 (84–100)	0.6
IL-18	RA–HS	0.93 (0.88–0.98)	1.1 × 10^−12^	246	77 (64–87)	98 (83–99)	0.75
Nuc. cfDNA	PsA–HS	0.9 (0.79–1)	2.8 × 10^−5^	1.54	90 (78–95)	89 (73–96)	0.78
Mt. cfDNA	PsA–HS	0.98 (0.95–1)	5.3 × 10^−7^	68	95 (85–98)	94 (80–99)	0.89
IL-18	PsA–HS	0.95 (0.9–1)	5 × 10^−11^	277	88 (75–94)	93 (78–98)	0.81
Mt. cfDNA	RA–AS	0.72 (0.6–0.83)	1.4 × 10^−3^	2903	87 (74–94)	56 (38–72)	0.43
MPO	RA–AS	0.89 (0.82–0.96)	1.1 × 10^−10^	1.3	71 (57–82)	100 (88–100)	0.71
H3Cit	SLE–RA	0.78 (0.66–0.91)	2 × 10^−4^	1.1	86 (74–93)	68 (50–82)	0.54
MPO	SLE–PsA	0.78 (0.64–0.92)	1.4 × 10^−3^	2	97 (87–99)	74 (56–86)	0.7
H3Cit	SLE–PsA	0.86 (0.75–0.96)	1.3 × 10^−5^	1	100 (93–100)	74 (56–86)	0.74
MPO	SLE–AS	0.91 (0.84–0.99)	1.5 × 10^−7^	2.1	94 (83–98)	74 (56–86)	0.68
H3Cit	SLE–AS	0.87 (0.79–0.95)	4.8 × 10^−7^	0.92	92 (81–97)	78 (61–89)	0.7

Note: Data are presented only for statistically significant binary logistic regression models.

**Table 3:** Results of two-way ANOVA showing the effects of bDMARDs therapy on NETosis markers.

Variable	Sum of Squares	df	F	*p*
Mitochondrial cfDNA				
C(Diagnosis)	3.26 × 10^8^	3	10.2	**0.00008**
C(Treatment)	2.33 × 10^7^	1	2.2	0.14
C(Diagnosis):C(Treatment)	1.47 × 10^8^	3	4.6	**0.004**
**H3cit**				
C(Diagnosis)	5.1	3	1.5	0.22
C(Treatment)	2.8	1	2.5	0.12
C(Diagnosis):C(Treatment)	14.0	3	4.2	**0.0069**
**MPO**				
C(Diagnosis)	44.3	3	3.0	0.054
C(Treatment)	47.4	1	9.5	**0.0024**
C(Diagnosis):C(Treatment)	238.4	3	16.0	**<0.0000001**

Note: The Treatment variable had two gradations: bDMARDs and csDMARDs.

**Table 4:** Primers for detection of genomic and mitochondrial cfDNA.

Gene	Primer	Sequence
*RP*: Human Ribonuclease P	Forward	5′-AGATTTGGACCTGCGAGCG-3′
	Reverse	5′-GAGCGGCTGTCTCCACAAGT-3′
	Probe	5′-HEX-TTCTGACCTGAAGGCTCTGCGCG-BHQ-1-3′
*MT-ND1*: NADH dehydrogenase subunit 1	Forward	5′-GAGCGATGGTGAGAGCTAAGGT-3′
	Reverse	5′-CCCTAAAACCCGCCACATCT-3′
	Probe	5′-Cy3.5-CCATCACCCTCTACATCACCGCCC-BHQ2-3′

## Data Availability

The original contributions presented in this study are included in the article/Supplementary Material. Further inquiries can be directed to the corresponding author.

## Supplementary Materials

The following supporting information can be downloaded at: https://www.mdpi.com/article/10.3390/ijms262412127/s1.

## Abbreviations

The following abbreviations are used in this manuscript:

RA	Rheumatoid arthritis
AS	Ankylosing spondylitis
PsA	Psoriatic arthritis
SLE	Systemic lupus erythematosus
HS	Healthy subjects
ssDNA	Single-stranded DNA
scDNA	Supercoiled DNA
DAS28	Disease Activity Score-28
ASDAS	AS disease activity score
BASDAI	Bath AS disease activity index
DAPSA	Disease activity in psoriatic arthritis
SELENA-SLEDAI	Safety of Estrogens in Lupus Erythematosus National Assessment—Systemic Lupus Erythematosus Disease Activity Index
cfDNA	Cell-free DNA
H3cit	Citrullinated Histone H3
MPO	Myeloperoxidase
IL	Interleukin
bDMARD	biological disease-modifying antirheumatic drugs
csDMARD	conventional synthetic disease-modifying antirheumatic drugs
PAD4	peptidyl arginine deaminase 4
CV	coefficient of variation
