# Supplementary material for: NETosis-Related Biomarkers in Systemic Lupus Erythematosus, Rheumatoid Arthritis, Psoriatic Arthritis and Ankylosing Spondylitis: A Comparative Study
Source: Int J Mol Sci. 2025 Dec 17;26(24):12127. doi: 10.3390/ijms262412127 (PMC12733578; doi:10.3390/ijms262412127)
Supplement: Supplementary file 1 [file ijms-26-12127-s001.zip › ijms-4016694-supplementary.pdf]

Supplementary materials

Article

NETosis-related biomarkers in systemic lupus erythematosus, rheumatoid arthritis, psoriatic arthritis and ankylosing spondylitis: a comparative study

Mark M. Melamud [1], Anna S. Tolmacheva [1], Alexey E. Sizikov [1,3], Nataliya A. Klyaus [4], Evgenii S. Zhuravlev [1], Grigory A. Stepanov [1], Georgy A. Nevinsky [1], Valentina N. Buneva [1,2,*], Evgeny A. Ermakov [1,2,*]

[1] Institute of Chemical Biology and Fundamental Medicine, Siberian Branch of the Russian Academy of Sciences, 630090 Novosibirsk, Russia

[2] Department of Natural Sciences, Novosibirsk State University, 630090 Novosibirsk, Russia

[3] Department of Rheumatology, Immunopathology Clinic, Research Institute of Fundamental and Clinical Immunology, Siberian Branch of the Russian Academy of Sciences, 630099 Novosibirsk, Russia

[4] Rheumatology and Immunopathology Research Laboratory, Almazov National Medical Research Centre, 197341 Saint Petersburg, Russia

* Correspondence: evgeny_ermakov@mail.ru (E.A.E.), buneva@niboch.nsc.ru (V.N.B.)

Table of contents

Table S1. Descriptive statistics for the analyzed groups. 4
Table S2. Parameters of the ANCOVA model for comparing total cfDNA in the analyzed groups. 7
Table S3. Post Hoc Comparisons results after ANCOVA for comparing total cfDNA in the analyzed groups. 8
Figure S1. Estimated marginal means in ANCOVA for comparing total cfDNA in the analyzed groups. 9
Table S4. Parameters of the ANCOVA model for comparing nuclear cfDNA in the analyzed groups. 10
Table S5. Post Hoc Comparisons results after ANCOVA for comparing nuclear cfDNA in the analyzed groups. 11
Figure S2. Estimated marginal means in ANCOVA for comparing nuclear cfDNA in the analyzed groups. 12
Table S6. Parameters of the ANCOVA model for comparing mitochondrial cfDNA in the analyzed groups. 13
Table S7. Post Hoc Comparisons results after ANCOVA for comparing mitochondrial cfDNA in the analyzed groups. 14
Figure S3. Estimated marginal means in ANCOVA for comparing mitochondrial cfDNA in the analyzed groups. 15
Table S8. Parameters of the ANCOVA model for comparing citrullinated histone H3 in the analyzed groups. 16
Table S9. Post Hoc Comparisons results after ANCOVA for comparing citrullinated histone H3 in the analyzed groups. 17
Figure S4. Estimated marginal means in ANCOVA for comparing citrullinated histone H3 in the analyzed groups. 18
Table S10. Parameters of the ANCOVA model for comparing myeloperoxidase in the analyzed groups. 19
Table S11. Post Hoc Comparisons results after ANCOVA for comparing myeloperoxidase in the analyzed groups. 20
Figure S5. Estimated marginal means in ANCOVA for comparing myeloperoxidase in the analyzed groups. 21
Table S12. Parameters of the ANCOVA model for comparing IL-18 in the analyzed groups. 22
Table S13. Post Hoc Comparisons results after ANCOVA for comparing IL-18 in the analyzed groups. 23
Figure S6. Estimated marginal means in ANCOVA for comparing IL-18 in the analyzed groups. 24
Figure S7. ROC curves for NETosis markers. 25

Table S14. Sex-dependent differences in the concentration of NETosis markers in patients with rheumatic diseases and healthy individuals. 26
Table S15. Myeloperoxidase and citrullinated histone H3 concentration in SLE patients depending on the presence of concomitant cardiovascular diseases (CVD). 28
Table S16. Mitochondrial cfDNA concentration in PsA patients depending on the disease activity score according to DAPSA. 28
Table S17. Mitochondrial cfDNA concentration in AS patients depending on the disease activity score according to ASDAS-CRP. 28
Table S18. Myeloperoxidase concentration in AS patients depending on the disease activity score according to BASDAI. 28
Table S19. Myeloperoxidase concentration in RA and mitochondrial cfDNA in PsA in patients in remission and active disease. 29
Table S20. Predictors of DAS28 score of RA patients in multiple regression analysis. 29
Table S21. Predictors of ASDAS-CRP score of AS patients in multiple regression analysis. 30
Table S22. Predictors of ASDAS-ESR score of AS patients in multiple regression analysis. 31
Table S23. Concentration of NETosis markers in patients with RA and PsA depending on the therapy received. 32

Table S1. Descriptive statistics for the analyzed groups.

Descriptives

	Group	Tot_cfDNA	mt_cf-DNA	n_cf-DNA	MPO	H3cit	IL-18	Age	Disease_duration
**N**	**AS**	53	45	44	49	50	52	53	53
	**HS**	73	19	19	48	48	48	73	0
	**PsA**	19	18	18	29	29	29	30	29
	**RA**	44	32	32	42	43	43	44	44
	**SLE**	23	19	19	19	23	23	23	23
**Missing**	**AS**	0	8	9	4	3	1	0	0
	**HS**	0	54	54	25	25	25	0	73
	**PsA**	10	11	11	0	0	0	0	0
	**RA**	0	12	12	2	1	1	0	0
	**SLE**	0	4	4	4	0	0	0	0
**Mean**	**AS**	44.4	1330	77.4	1.13	0.701	463	44.9	16.4
	**HS**	17.8	37.6	0.713	1.22	0.803	221	37.8	NaN
	**PsA**	16.0	1424	3.18	1.63	0.730	448	45.1	13.1
	**RA**	40.8	4678	6.32	2.83	1.23	443	49.5	9.83
	**SLE**	31.1	569	6.79	5.58	1.78	564	44.1	8.33
**Median**	**AS**	19.6	734	3.93	0.877	0.657	420	46	12.0
	**HS**	10.4	35.2	0.00	1.21	0.686	212	37	NaN
	**PsA**	16.1	756	2.91	1.62	0.760	431	42	10.0

Descriptives

	Group	Tot_cfDNA	mt_cf-DNA	n_cf-DNA	MPO	H3cit	IL-18	Age	Disease_duration
	**RA**	13.3	3258	4.71	1.88	0.803	444	51.0	8.00
	**SLE**	25.5	598	5.83	3.23	1.44	461	44	6.00
**Standard deviation**	**AS**	102	1460	265	0.741	0.227	281	12.1	11.3
	**HS**	16.2	22.5	1.36	0.570	0.563	70.1	12.8	NaN
	**PsA**	6.77	1431	1.63	0.297	0.171	126	12.9	11.2
	**RA**	125	5098	6.75	2.60	1.88	148	12.6	7.08
	**SLE**	22.4	268	2.74	4.92	1.12	361	16.0	7.49
**IQR**	**AS**	41.3	1997	6.00	0.807	0.239	271	14.0	14.0
	**HS**	17.9	23.6	0.579	0.823	0.335	57.3	17.0	NaN
	**PsA**	7.56	2548	1.89	0.237	0.297	102	20.0	18.0
	**RA**	20.5	6763	3.47	1.01	0.354	257	21.0	10.3
	**SLE**	17.8	376	4.73	6.56	1.56	382	21.5	9.00
**Minimum**	**AS**	0.00	1.99	0.396	0.485	0.409	114	18	1.00
	**HS**	0.608	14.2	0.00	0.00	0.371	100	22	NaN
	**PsA**	6.26	44.4	0.395	0.925	0.393	206	26	1.00
	**RA**	0.850	7.70	0.390	1.37	0.453	191	27	0.500
	**SLE**	12.6	118	3.79	1.05	0.558	184	19	0.500
**Maximum**	**AS**	749	7117	1494	4.68	1.55	1572	76	50.0
	**HS**	69.8	112	4.63	2.97	4.34	535	77	NaN
	**PsA**	32.4	3832	6.13	2.61	0.998	756	71	35.0

Descriptives

	Group	Tot_cfDNA	mt_cf-DNA	n_cf-DNA	MPO	H3cit	IL-18	Age	Disease_duration
	**RA**	834	18207	32.4	14.0	11.7	763	69	24.0
	**SLE**	90.9	971	12.4	20.2	4.49	1847	72	27.0
**Shapiro-Wilk W**	**AS**	0.311	0.816	0.320	0.734	0.843	0.782	0.983	0.927
	**HS**	0.786	0.800	0.597	0.977	0.449	0.842	0.910	NaN
	**PsA**	0.943	0.815	0.963	0.915	0.954	0.972	0.946	0.878
	**RA**	0.272	0.824	0.593	0.527	0.337	0.955	0.947	0.908
	**SLE**	0.774	0.954	0.882	0.826	0.880	0.805	0.957	0.852
**Shapiro-Wilk p**	**AS**	<.001	<.001	<.001	<.001	<.001	<.001	0.669	0.003
	**HS**	<.001	0.001	<.001	0.447	<.001	<.001	<.001	NaN
	**PsA**	0.301	0.003	0.669	0.023	0.231	0.629	0.145	0.003
	**RA**	<.001	<.001	<.001	<.001	<.001	0.089	0.042	0.002
	**SLE**	<.001	0.468	0.023	0.003	0.010	<.001	0.396	0.003
**25th percentile**	**AS**	10.9	297	2.80	0.658	0.557	289	38.0	8.00
	**HS**	6.03	19.3	0.00	0.797	0.565	187	27.0	NaN
	**PsA**	11.0	291	2.29	1.53	0.580	395	34.0	3.00
	**RA**	6.36	517	3.19	1.63	0.657	306	38.5	4.75
	**SLE**	15.0	380	4.49	1.81	0.971	342	32.5	3.00
**75th percentile**	**AS**	52.2	2294	8.80	1.46	0.797	560	52.0	22.0
	**HS**	23.9	42.9	0.579	1.62	0.900	244	44.0	NaN
	**PsA**	18.5	2839	4.18	1.76	0.877	496	54.0	21.0

Descriptives

	Group	Tot_cfDNA	mt_cf-DNA	n_cf-DNA	MPO	H3cit	IL-18	Age	Disease_duration
	**RA**	26.9	7279	6.66	2.64	1.01	563	59.5	15.0
	**SLE**	32.7	756	9.22	8.37	2.53	724	54.0	12.0

Table S2. Parameters of the ANCOVA model for comparing total cfDNA in the analyzed groups.

ANCOVA - Tot_cfDNA

	Sum of Squares	df	Mean Square	F	p	η^2	η^2p	ω^2
**Overall model**	14.005	7	2.001	2.471	**0.019**			
**Age**	0.598	1	0.598	0.690	0.407	0.003	0.003	-0.001
**Group**	12.984	4	3.246	3.748	**0.006**	0.068	0.068	0.050
**Dis_Duration**	0.252	1	0.252	0.291	0.590	0.001	0.001	-0.003
**Sex**	0.171	1	0.171	0.197	0.657	0.001	0.001	-0.004
**Residuals**	176.687	204	0.866					

Table S3. Post Hoc Comparisons results after ANCOVA for comparing total cfDNA in the analyzed groups.

Post Hoc Comparisons - Group

**Comparison**							
**Group**		**Group**	**Mean Difference**	**SE**	**df**	**t**	p_{tukey}
**AS**	-	**HS**	0.6566	0.223	204	2.944	**0.029**
	-	**PsA**	0.3890	0.256	204	1.517	0.552
	-	**RA**	0.4714	0.211	204	2.235	0.171
	-	**SLE**	-0.0505	0.262	204	-0.193	1.000
**HS**	-	**PsA**	-0.2676	0.283	204	-0.946	0.878
	-	**RA**	-0.1852	0.200	204	-0.927	0.886
	-	**SLE**	-0.7071	0.239	204	-2.958	**0.028**
**PsA**	-	**RA**	0.0824	0.265	204	0.311	0.998
	-	**SLE**	-0.4395	0.302	204	-1.457	0.591
**RA**	-	**SLE**	-0.5219	0.244	204	-2.140	0.207

Note. Comparisons are based on estimated marginal means

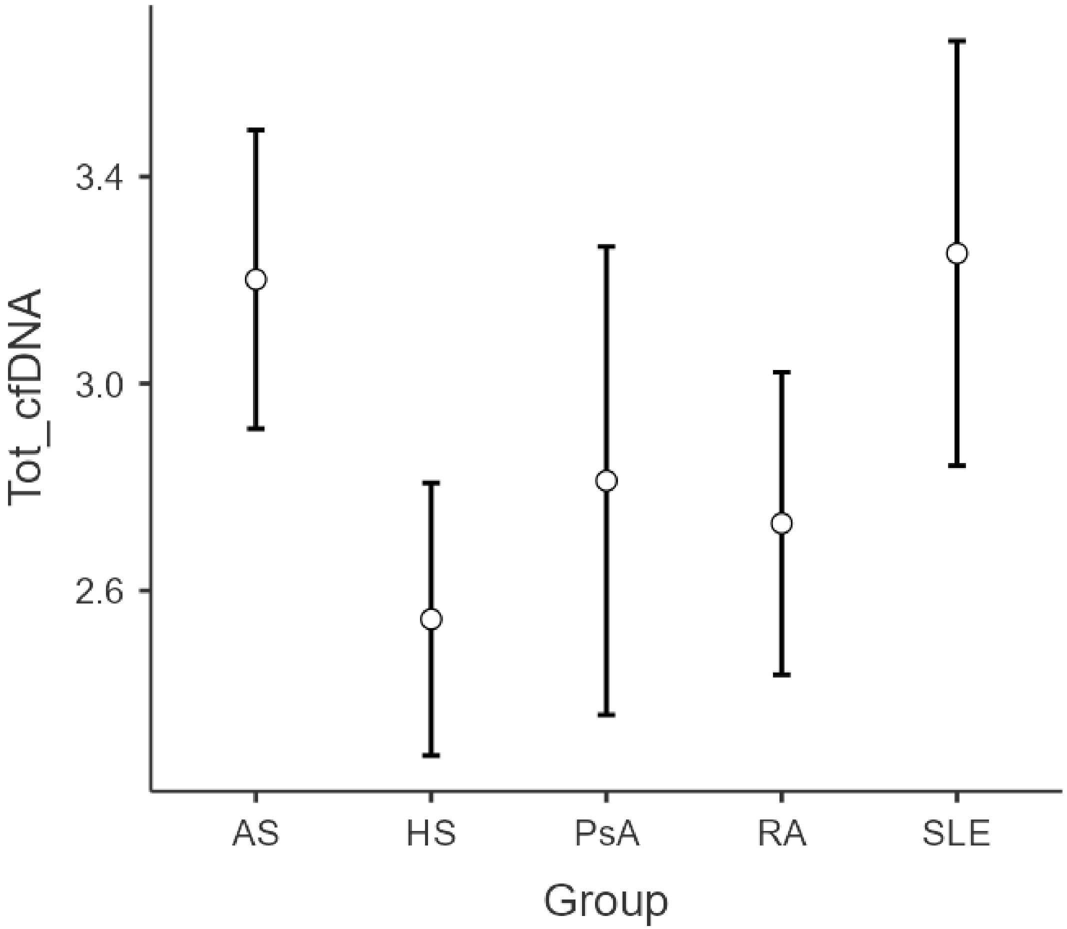

Figure S1. Estimated marginal means in ANCOVA for comparing total cfDNA in the analyzed groups.

Table S4. Parameters of the ANCOVA model for comparing nuclear cfDNA in the analyzed groups.

ANCOVA - n_cf-DNA

	Sum of Squares	df	Mean Square	F	p	η^2	$\eta^2 p$	ω^2
**Overall model**	46.742	7	6.677	7.449	**<.001**			
**Age**	1.702	1	1.702	1.704	0.194	0.010	0.014	0.004
**Group**	44.138	4	11.035	11.047	**<.001**	0.259	0.263	0.234
**Dis_Duration**	0.519	1	0.519	0.520	0.472	0.003	0.004	-0.003
**Sex**	0.382	1	0.382	0.382	0.537	0.002	0.003	-0.004
**Residuals**	123.858	124	0.999					

Table S5. Post Hoc Comparisons results after ANCOVA for comparing nuclear cfDNA in the analyzed groups.

Post Hoc Comparisons - Group

**Comparison**							
**Group**		**Group**	**Mean Difference**	**SE**	**df**	**t**	p_{tukey}
**AS**	-	**HS**	2.047	0.357	124	5.734	**<.001**
	-	**PsA**	0.710	0.298	124	2.384	0.127
	-	**RA**	0.331	0.281	124	1.179	0.764
	-	**SLE**	0.125	0.341	124	0.366	0.996
**HS**	-	**PsA**	-1.337	0.386	124	-3.461	**0.007**
	-	**RA**	-1.715	0.334	124	-5.136	**<.001**
	-	**SLE**	-1.922	0.352	124	-5.455	**<.001**
**PsA**	-	**RA**	-0.379	0.308	124	-1.229	0.734
	-	**SLE**	-0.585	0.351	124	-1.670	0.456
**RA**	-	**SLE**	-0.207	0.298	124	-0.693	0.958

Note. Comparisons are based on estimated marginal means

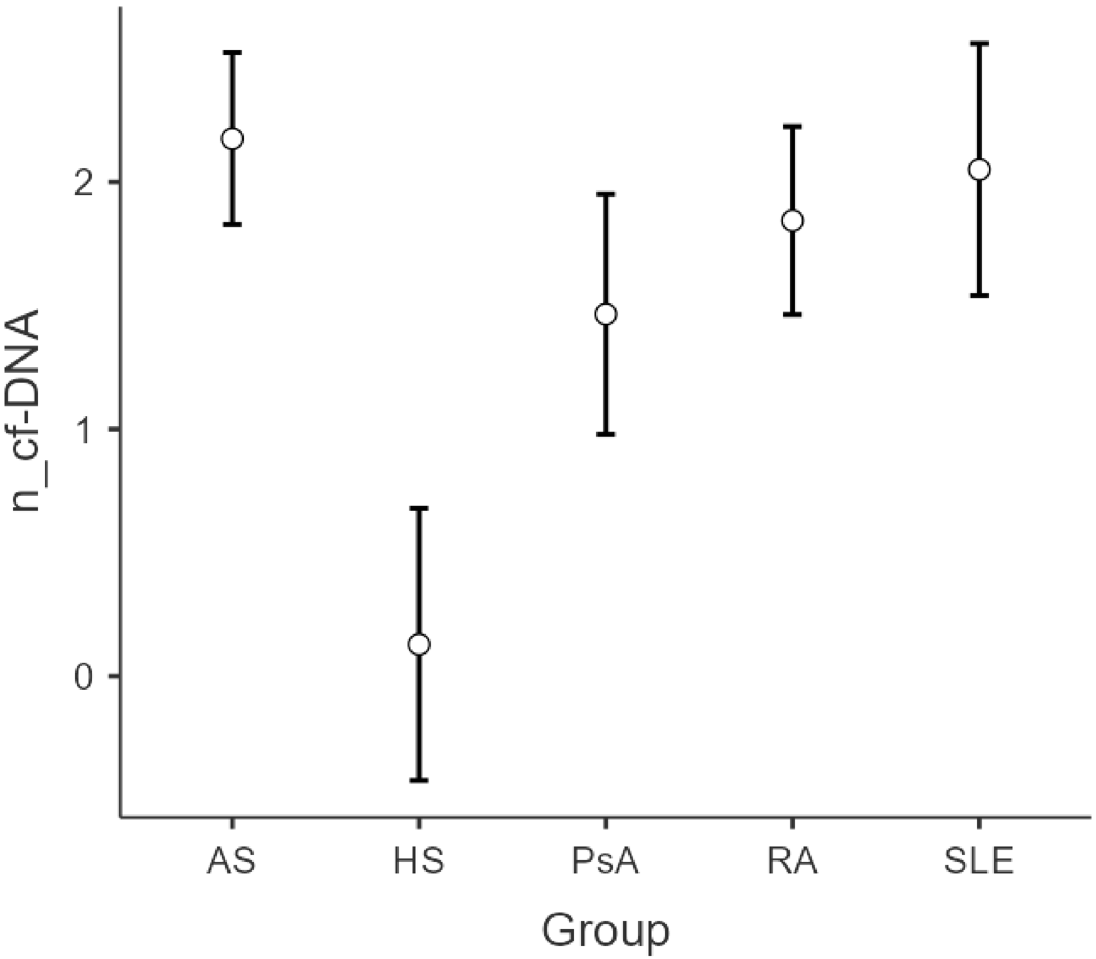

Figure S2. Estimated marginal means in ANCOVA for comparing nuclear cfDNA in the analyzed groups.

Table S6. Parameters of the ANCOVA model for comparing mitochondrial cfDNA in the analyzed groups.

ANCOVA - mt_cf-DNA

	Sum of Squares	df	Mean Square	F	p	η^2	$\eta^2 p$	ω^2
**Overall model**	132.0536	7	18.8648	10.5378	**<.001**			
**Age**	0.7093	1	0.7093	0.2699	0.604	0.002	0.002	-0.004
**Group**	131.2145	4	32.8036	12.4830	**<.001**	0.285	0.285	0.261
**Dis_Duration**	0.0506	1	0.0506	0.0193	0.890	0.000	0.000	-0.006
**Sex**	0.0792	1	0.0792	0.0301	0.862	0.000	0.000	-0.006
**Residuals**	328.4841	125	2.6279					

Table S7. Post Hoc Comparisons results after ANCOVA for comparing mitochondrial cfDNA in the analyzed groups.

Post Hoc Comparisons - Group

Comparison							
**Group**		**Group**	**Mean Difference**	**SE**	**df**	**t**	**p_{tukey}**
**AS**	**-**	**HS**	2.4719	0.572	125	4.320	**<.001**
	**-**	**PsA**	-0.3587	0.478	125	-0.751	0.944
	**-**	**RA**	-1.3343	0.445	125	-2.998	**0.027**
	**-**	**SLE**	-0.0817	0.542	125	-0.151	1.000
**HS**	**-**	**PsA**	-2.8307	0.626	125	-4.520	**<.001**
	**-**	**RA**	-3.8062	0.542	125	-7.027	**<.001**
	**-**	**SLE**	-2.5536	0.571	125	-4.471	**<.001**
**PsA**	**-**	**RA**	-0.9755	0.499	125	-1.955	0.294
	**-**	**SLE**	0.2771	0.567	125	0.489	0.988
**RA**	**-**	**SLE**	1.2526	0.483	125	2.592	0.078

Note. Comparisons are based on estimated marginal means

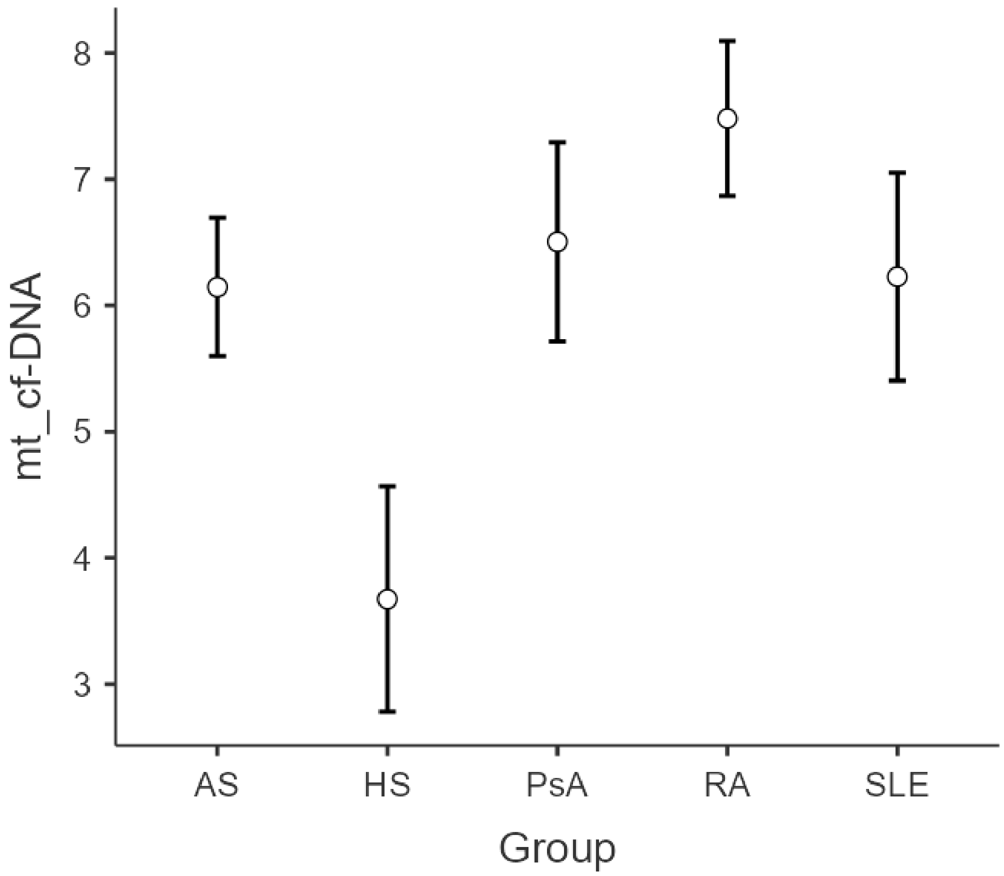

Figure S3. Estimated marginal means in ANCOVA for comparing mitochondrial cfDNA in the analyzed groups.

Table S8. Parameters of the ANCOVA model for comparing citrullinated histone H3 in the analyzed groups.

ANCOVA - H3cit

	Sum of Squares	df	Mean Square	F	p	η^2	η^2p	ω^2
**Overall model**	3.0104	7	0.4301	7.826	**<.001**			
**Age**	0.0133	1	0.0133	0.200	0.655	0.001	0.001	-0.003
**Group**	2.8236	4	0.7059	10.606	**<.001**	0.184	0.187	0.166
**Dis_Duration**	0.0849	1	0.0849	1.276	0.260	0.006	0.007	0.001
**Sex**	0.0886	1	0.0886	1.332	0.250	0.006	0.007	0.001
**Residuals**	12.3129	185	0.0666					

Table S9. Post Hoc Comparisons results after ANCOVA for comparing citrullinated histone H3 in the analyzed groups.

Post Hoc Comparisons - Group

Comparison							
**Group**		**Group**	**Mean Difference**	**SE**	**df**	**t**	p_{tukey}
**AS**	-	**HS**	-0.06298	0.0651	185	-0.9676	0.869
	-	**PsA**	-0.00529	0.0637	185	-0.0831	1.000
	-	**RA**	-0.15355	0.0598	185	-2.5691	0.080
	-	**SLE**	-0.41568	0.0733	185	-5.6681	**<.001**
**HS**	-	**PsA**	0.05769	0.0681	185	0.8476	0.915
	-	**RA**	-0.09057	0.0590	185	-1.5339	0.542
	-	**SLE**	-0.35270	0.0703	185	-5.0196	**<.001**
**PsA**	-	**RA**	-0.14826	0.0633	185	-2.3411	0.137
	-	**SLE**	-0.41038	0.0734	185	-5.5891	**<.001**
**RA**	-	**SLE**	-0.26212	0.0681	185	-3.8468	0.002

Note. Comparisons are based on estimated marginal means

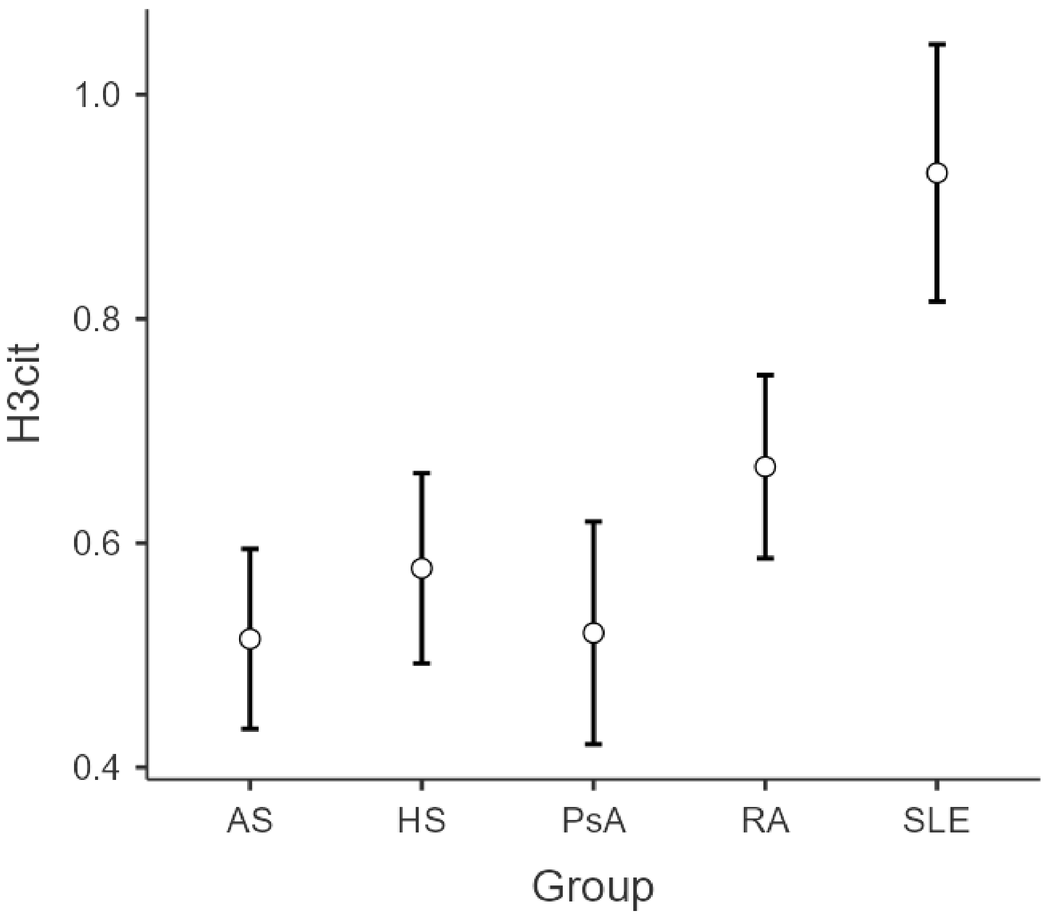

Figure S4. Estimated marginal means in ANCOVA for comparing citrullinated histone H3 in the analyzed groups.

Table S10. Parameters of the ANCOVA model for comparing myeloperoxidase in the analyzed groups.

ANCOVA - MPO

	Sum of Squares	df	Mean Square	F	p	η^2	η^2p	ω^2
**Overall model**	15.6122	7	2.2303	19.11903	**<.001**			
**Age**	0.3484	1	0.3484	2.64495	0.106	0.009	0.014	0.005
**Group**	15.1650	4	3.7913	28.78033	**<.001**	0.386	0.390	0.371
**Dis_Duration**	6.04e-4	1	6.04e-4	0.00459	0.946	0.000	0.000	-0.003
**Sex**	0.0981	1	0.0981	0.74466	0.389	0.002	0.004	-0.001
**Residuals**	23.7116	180	0.1317					

Table S11. Post Hoc Comparisons results after ANCOVA for comparing myeloperoxidase in the analyzed groups.

Post Hoc Comparisons - Group

**Comparison**							
**Group**		**Group**	**Mean Difference**	**SE**	**df**	**t**	p_{tukey}
**AS**	**-**	**HS**	-0.0574	0.0917	180	-0.625	0.971
	**-**	**PsA**	-0.2708	0.0897	180	-3.018	**0.024**
	**-**	**RA**	-0.5164	0.0841	180	-6.144	**<.001**
	**-**	**SLE**	-0.9690	0.1090	180	-8.892	**<.001**
**HS**	**-**	**PsA**	-0.2134	0.0959	180	-2.224	0.175
	**-**	**RA**	-0.4590	0.0832	180	-5.516	**<.001**
	**-**	**SLE**	-0.9116	0.1045	180	-8.723	**<.001**
**PsA**	**-**	**RA**	-0.2456	0.0893	180	-2.749	0.051
	**-**	**SLE**	-0.6982	0.1091	180	-6.400	**<.001**
**RA**	**-**	**SLE**	-0.4525	0.1021	180	-4.431	**<.001**

Note. Comparisons are based on estimated marginal means

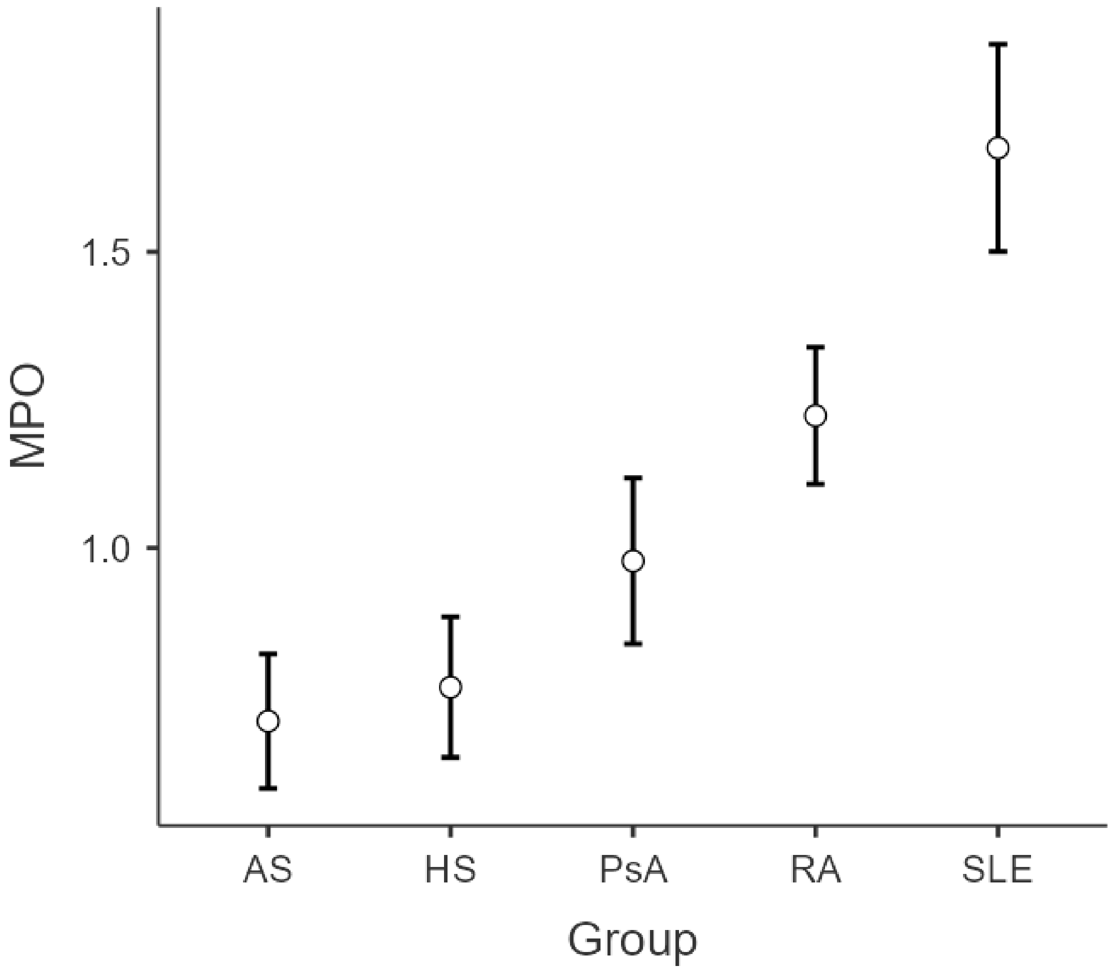

Figure S5. Estimated marginal means in ANCOVA for comparing myeloperoxidase in the analyzed groups.

Table S12. Parameters of the ANCOVA model for comparing IL-18 in the analyzed groups.

ANCOVA - IL-18

	Sum of Squares	df	Mean Square	F	p	η^2	η^2p	ω^2
**Overall model**	14.15379	7	2.02197	15.4759	**<.001**			
**Age**	0.06473	1	0.06473	0.3851	0.536	0.001	0.002	-0.002
**Group**	13.70538	4	3.42635	20.3854	**<.001**	0.301	0.304	0.285
**Dis_Duration**	0.00609	1	0.00609	0.0362	0.849	0.000	0.000	-0.004
**Sex**	0.37758	1	0.37758	2.2465	0.136	0.008	0.012	0.005
**Residuals**	31.43065	187	0.16808					

Table S13. Post Hoc Comparisons results after ANCOVA for comparing IL-18 in the analyzed groups.

Post Hoc Comparisons - Group

Comparison							
**Group**		**Group**	**Mean Difference**	**SE**	**df**	**t**	**p_{tukey}**
**AS**	**-**	**HS**	0.6075	0.1025	187	5.926	**<.001**
	**-**	**PsA**	-0.1096	0.1003	187	-1.092	0.810
	**-**	**RA**	-0.0689	0.0937	187	-0.735	0.948
	**-**	**SLE**	-0.2473	0.1156	187	-2.139	0.208
**HS**	**-**	**PsA**	-0.7171	0.1080	187	-6.641	**<.001**
	**-**	**RA**	-0.6763	0.0937	187	-7.216	**<.001**
	**-**	**SLE**	-0.8548	0.1115	187	-7.669	**<.001**
**PsA**	**-**	**RA**	0.0407	0.1005	187	0.405	0.994
	**-**	**SLE**	-0.1377	0.1167	187	-1.180	0.763
**RA**	**-**	**SLE**	-0.1785	0.1082	187	-1.650	0.468

Note. Comparisons are based on estimated marginal means

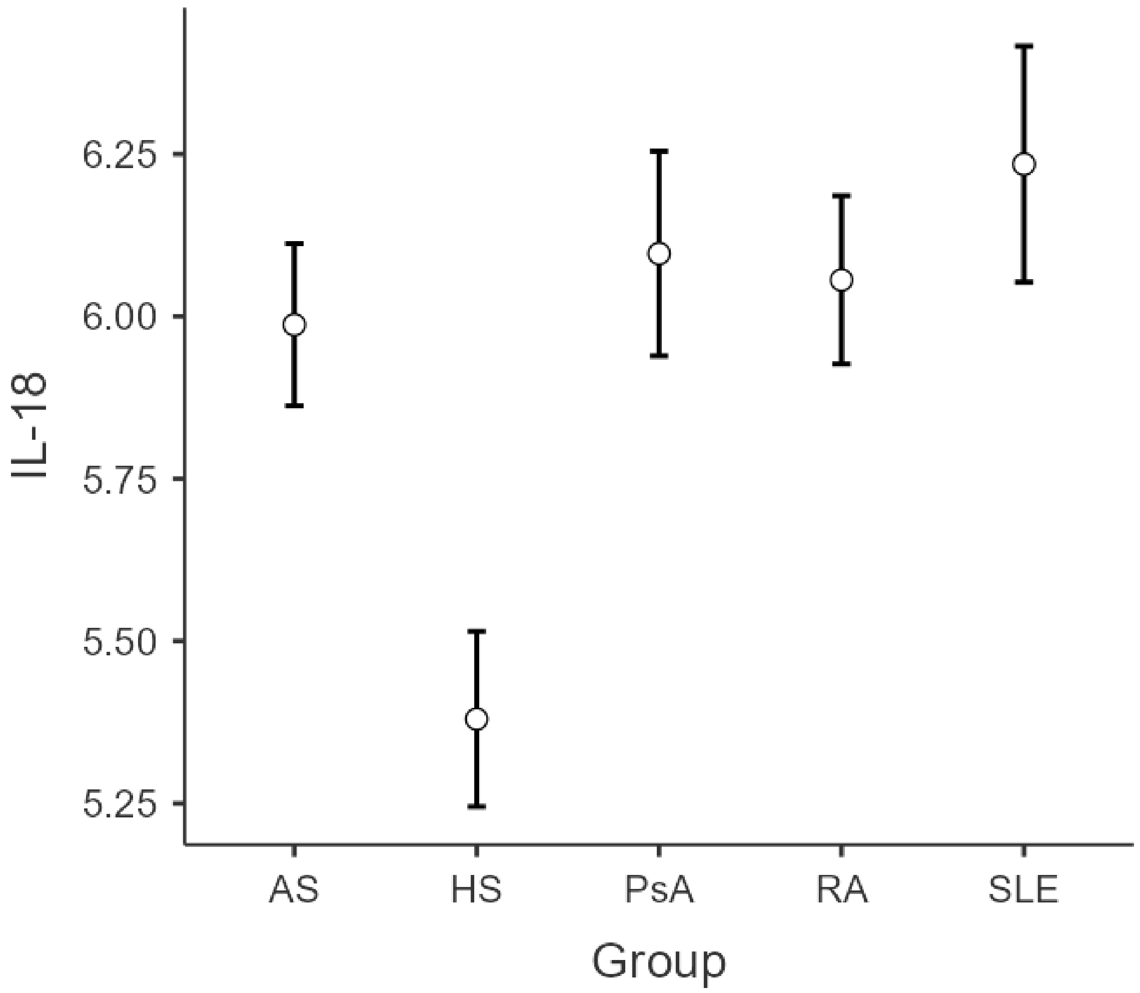

Figure S6. Estimated marginal means in ANCOVA for comparing IL-18 in the analyzed groups.

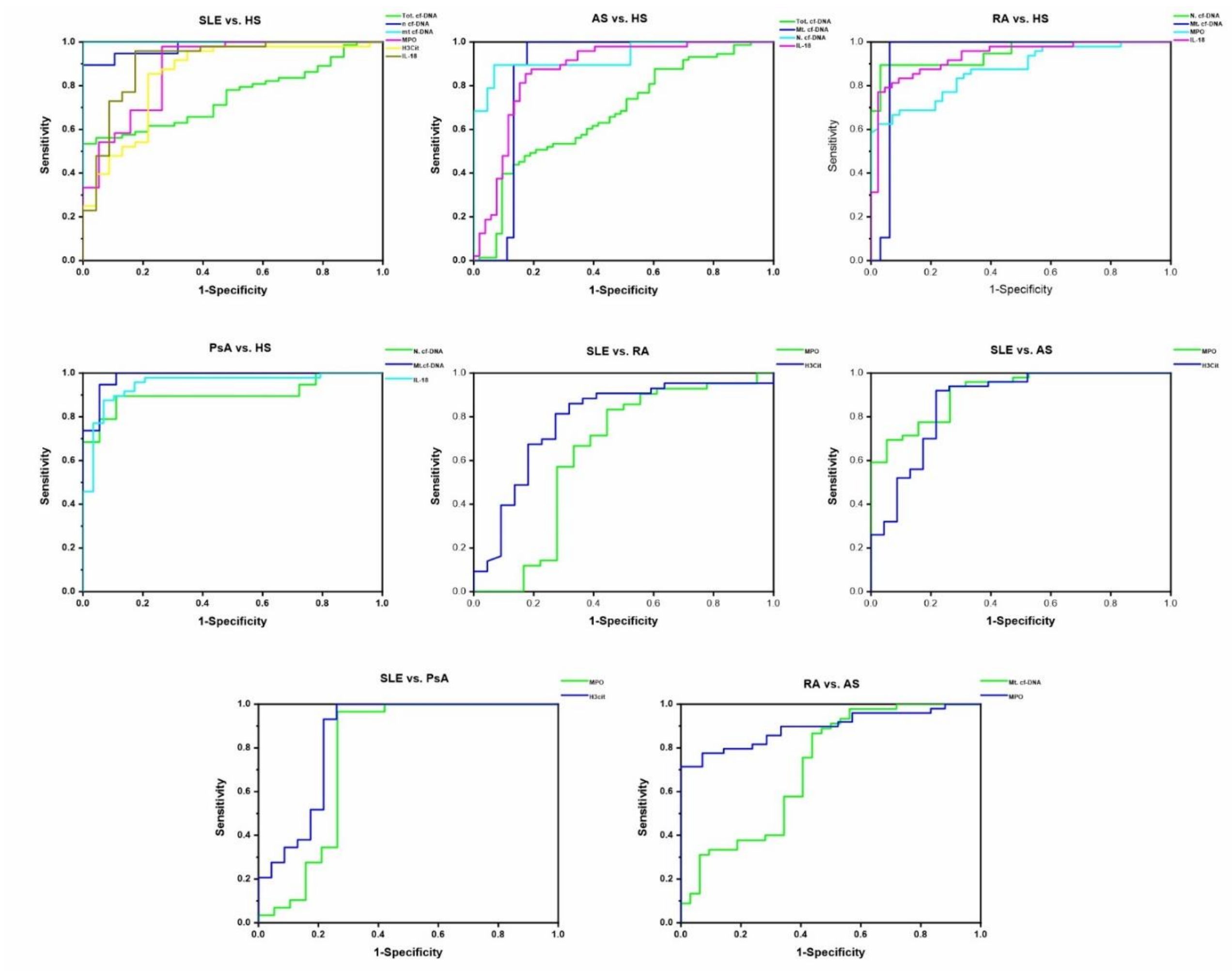

Figure S7. ROC curves for NETosis markers.

Table S14. Sex-dependent differences in the concentration of NETosis markers in patients with rheumatic diseases and healthy individuals.

Marker	Sex	Median	Q1	Q3	P-value
**RA**					
**Tot. cfDNA**	F	15.9	10.7	39.7	0.5
	M	14.3	8	52.2	
**Mt. cfDNA**	F	3307	491	8715	0.31
	M	2296	350	4702	
**Nuc. cfDNA**	F	4.7	3	10.5	0.84
	M	5.2	1.5	6.8	
**MPO**	F	1.9	1.6	2.2	0.07
	M	2.6	1.9	2.8	
**H3cit**	F	0.79	0.66	1.02	0.53
	M	0.8	0.63	0.97	
**IL-18**	F	365	303	488	**4.90E-04**
	M	571	480	671	
**AS**					
**Tot. cfDNA**	F	24.8	13.3	53.7	0.12
	M	15.6	9.9	46.1	
**Mt. cfDNA**	F	619	216	3125	1
	M	698	280	2107	
**Nuc. cfDNA**	F	2.8	1.2	3.4	0.06
	M	4.4	2.4	7.9	
**MPO**	F	0.77	0.64	1.8	0.49
	M	0.96	0.69	1.42	
**H3cit**	F	0.66	0.48	0.81	0.19
	M	0.72	0.58	0.84	
**IL-18**	F	302	260	594	0.26

	M	439	346	544	
**PsA**					
**Tot. cfDNA**	F	14.6	8.8	19.9	**0.04**
	M	18.7	16.8	27.4	
**Mt. cfDNA**	F	1562	166	3357	0.77
	M	412	268	3832	
**Nuc. cfDNA**	F	3.4	2.3	5	0.56
	M	3.1	0.8	4.4	
**MPO**	F	1.7	1.58	1.83	**0.03**
	M	1.56	1.42	1.61	
**H3cit**	F	0.82	0.62	0.9	**0.049**
	M	0.64	0.53	0.74	
**IL-18**	F	438	395	540	0.31
	M	403	371	535	
**HS**					
**Tot. cfDNA**	F	9.1	5.5	21.9	0.18
	M	13.3	7.4	28	
**Mt. cfDNA**	F	33	18	39	**0.007**
	M	50	42	87	
**Nuc. cfDNA**	F	0	0	0.49	0.76
	M	0	0	2.7	
**MPO**	F	1.16	0.71	1.62	0.62
	M	1.3	0.88	1.71	
**H3cit**	F	0.62	0.56	0.87	0.41
	M	0.86	0.55	0.94	
**IL-18**	F	196	186	239	0.35
	M	223	183	250	

Note: All patients with SLE were female, so no analysis was performed for SLE.

Table S15. Myeloperoxidase and citrullinated histone H3 concentration in SLE patients depending on the presence of concomitant cardiovascular diseases (CVD).

Marker	No CVD (14)	CVD (9)	P-value*
**MPO**	2.6 (1.4, 4.4)	8.7 (5.8, 10.8)	**0.008**
**H3cit**	1.1 (0.7, 1.8)	2.2 (1.5, 2.9)	**0.02**

* Mann-Whitney test. Data are presented as median (Q1–Q3).

Table S16. Mitochondrial cfDNA concentration in PsA patients depending on the disease activity score according to DAPSA.

Marker	DAPSA≤14 (No-Low activity) (n=14)	DAPSA>14 (Moderate-High activity) (n=16)	P-value*
**Mt. cfDNA**	340 (110, 1247)	2334 (644, 3624)	**0.03**

* Mann-Whitney test. Data are presented as median (Q1–Q3).

Table S17. Mitochondrial cfDNA concentration in AS patients depending on the disease activity score according to ASDAS-CRP.

Marker	ASDAS-CRP≤2.1 (No–Low activity) (n=32)	ASDAS-CRP>2.1 (High –Very High activity) (n=19)	P-value*
**Mt. cf-DNA**	678 (248, 1506)	2213 (460, 3670)	**0.049**

* Mann-Whitney test. Data are presented as median (Q1–Q3).

Table S18. Myeloperoxidase concentration in AS patients depending on the disease activity score according to BASDAI.

Marker	BASDAI≤3 (Low activity) (n=39)	BASDAI>3 (Moderate activity) (n=15)	P-value*
**MPO**	0.84 (0.63, 1.17)	1.58 (0.77, 1.9)	**0.02**

* Mann-Whitney test. Data are presented as median (Q1–Q3).

Table S19. Myeloperoxidase concentration in RA and mitochondrial cfDNA in PsA in patients in remission and active disease.

Marker	Remission	Active disease/exacerbation	P-value*
**MPO in RA**	1.60 (1.52, 2.04)	2.16 (1.82, 2.91)	**0.02**
**Mt. cf-DNA in PsA**	340 (1.9, 1247)	2334 (644, 3624)	**0.03**

* Mann-Whitney test. Data are presented as median (Q1–Q3).

Table S20. Predictors of DAS28 score of RA patients in multiple regression analysis.

Variable	β	Standard Error	t	p	Model
Constant	0.3072	0.231	1.329	0.191	R^2_{adj}=0.398; F=5.553; **p=5.85e-5**
Tot. cfDNA	-0.0641	0.048	-1.349	0.185	
Mt. cfDNA	0.0096	0.022	0.437	0.665	
Nuc. cfDNA	-0.0298	0.031	-0.953	0.346	
MPO	0.1201	0.166	0.723	0.474	
H3cit	-0.1745	0.381	-0.458	0.649	
IL-18	0.0326	0.087	0.375	0.71	
CRP	0.1908	0.054	3.518	**0.001**	
ESR	0.0303	0.058	0.525	0.603	

Note: Significant predictors ($p < 0.05$) are highlighted in bold.

Table S21. Predictors of ASDAS-CRP score of AS patients in multiple regression analysis.

Variable	β	Standard Error	t	p	Model
Constant	0.3072	0.231	1.329	0.191	R^2_{adj}=0.316; F=3.883; **p=0.0017**
Tot. cfDNA	-0.0641	0.048	-1.349	0.185	
Mt. cfDNA	0.0096	0.022	0.437	0.665	
Nuc. cfDNA	-0.0298	0.031	-0.953	0.346	
MPO	0.1201	0.166	0.723	0.474	
H3cit	-0.1745	0.381	-0.458	0.649	
IL-18	0.0326	0.087	0.375	0.71	
CRP	0.1908	0.054	3.518	**0.001**	
ESR	0.0303	0.058	0.525	0.603	

Note: Significant predictors (p < 0.05) are highlighted in bold.

Table S22. Predictors of ASDAS-ESR score of AS patients in multiple regression analysis.

Variable	β	Standard Error	t	p	Model
Constant	0.3346	0.268	1.249	0.218	R^2_{adj}=0.264; F=3.329; **p=0.0046**
Tot. cfDNA	-0.0636	0.051	-1.251	0.217	
Mt. cfDNA	0.012	0.027	0.445	0.658	
Nuc. cfDNA	-0.0409	0.038	-1.082	0.285	
MPO	0.113	0.194	0.583	0.563	
H3cit	-0.2461	0.453	-0.543	0.59	
IL-18	-0.0021	0.103	-0.02	0.984	
CRP	0.0337	0.066	0.513	0.61	
ESR	0.2209	0.069	3.211	**0.002**	

Note: Significant predictors ($p < 0.05$) are highlighted in bold.

Table S23. Concentration of NETosis markers in patients with RA and PsA depending on the therapy received.

Marker	bDMARDs	csDMARDs or glucocorticoid drugs	P-value*
		**RA**	
**Mt. cfDNA**	703 (405, 5183)	5711 (1996, 11720)	**0.021**
**H3cit**	0.70 (0.60, 0.99)	0.70 (0.57, 1.02)	0.917
**MPO**	2.25 (1.62, 3.52)	1.94 (1.77, 2.61)	0.386
		**PsA**	
**Mt. cfDNA**	756 (203, 3685)	1361 (569, 2739)	0.925
**H3cit**	0.67 (0.56, 0.75)	0.82 (0.80, 0.90)	**0.016**
**MPO**	1.57 (1.42, 1.81)	1.70 (1.56, 1.76)	0.406

* Mann-Whitney test. Data are presented as median (Q1–Q3). bDMARDs – biological disease-modifying antirheumatic drugs. csDMARDs – conventional synthetic disease-modifying antirheumatic drugs.
